# Genome Wide Analysis of Sex Difference in Gene Expression Profiles of Bone Formations Using *sfx* Mice and BXD RI Strains

**DOI:** 10.1155/2014/584910

**Published:** 2014-07-14

**Authors:** Yue Huang, Xiaodong Zhu, Lishi Wang, Xiaoyun Liu, Lu Lu, Weikuan Gu, Yan Jiao

**Affiliations:** ^1^Department of Orthopedic Surgery and BME, University of Tennessee Health Science Center, 956 Court Avenue, Memphis, TN 38163, USA; ^2^Mudanjiang Medical College, Tongxiang Road, Aimin District, Mudanjiang City, Heilongjiang 157001, China; ^3^Department of Basic Research, Inner Mongolia Medical College, Inner Mongolia 010110, China; ^4^Department of Anatomy and Neurobiology, University of Tennessee Health Science Center, Memphis, TN 38163, USA

## Abstract

The objective of this study is to identify sex differentially expressed genes in bone using a mouse model of spontaneous fracture, *sfx*, which lacks the gene for L-gulonolactone oxidase (*Gulo*), a key enzyme in the ascorbic acid (AA) synthesis pathway. We first identified the genes that are differentially expressed in the femur between female and male in *sfx* mice. We then analyzed the potential gene network among those differentially expressed genes with whole genome expression profiles generated using spleens of female and male mice of a total of 67 BXD (C57BL/6J X DBA/2J) recombinant inbred (RI) and other strains. Our result indicated that there was a sex difference in the whole genome profiles in *sfx* mice as measured by the proportion of up- and downregulated genes. Several genes in the pathway of bone development are differentially expressed between the male and female of *sfx* mice. Comparison of gene network of up- and downregulated bone relevant genes also suggests a sex difference.

## 1. Introduction

In humans, gender difference in vitamin C (VC) requirement has been suggested by several studies [1–3]. However, the mechanism of such a difference needs to be explored. One issue is the skeletal difference between men and women. VC is essential for the skeletal development. It is not clear whether the different VC requirement is due to the gender skeletal difference. Recently study on the 970 human skeletons from mass* burials* dating to the height of the famine in Kilkenny City [4] provided an opportunity to study the skeletal manifestations of scurvy—a disease that became widespread at this time due to the sudden lack of VC. Geber and Murphy found that a sex and stature bias is evident among adults in which males and taller individuals displayed statistically significantly higher levels of scorbutic lesions. Thus the study provided evidence to support an investigation on the gender difference when lacking VC in humans. In animal study, a sex difference has also been evidenced [5, 6].

Previously, we have been usinga spontaneous bone fracture (*sfx*) mouse [7] for the studies of the effect of VC. We first discovered that this* sfx *model lacks the gene for L-gulonolactone oxidase (*Gulo*), a key enzyme in the ascorbic acid (AA) synthesis pathway [8] confirmed to be a model of ascorbic acid deficiency (scurvy). We then examined gene expression profiles of three tissues between* sfx *and the wild type [9, 10]. The other set of the widely used animals for study of gene expression profiles is the largest and well-defined recombinant inbred (RI) strains, the RI strains derived from C57BL/6J (B6) and DBA/2J (D2) progenitor strains [11]. The BXD RI strains are a well-characterized set of strains for which a remarkable variety of phenotype data and gene expression profiles have already been acquired. Data of phenotypes and genotypes and gene expressions can be easily obtained from the GeneNetwork page: http://www.genenetwork.org. In this study, we investigated the gene expression profiles between female and male mice using the* sfx *mouse model and BXD RI strains.

## 2. Materials and Methods

### 2.1. Animals

Two sets of mice were used. The first set was* sfx *mice and their wild types (Wt). Three females and three males of 6-week-old* sfx *and wild type mice were used for the experiment. The mice were handled according to a protocol previously described [8, 12]. The second set of mice was the BXD mice. The BXD set of RI strains were derived by crossing C57BL/6J (B6) and DBA/2J (D2) and inbreeding progeny for more than 20 generations [11]. The gene expression profiles of BXD mice were provided by Dr. Williams' laboratory. Experimental procedures for this study were approved by the Institutional Animal Care and Use Committee at UTHSC and at VAMC in Memphis.

### 2.2. Procedure of Generation of Gene Expression Profiles* sfx *Mice

Total RNA was isolated from the femurs of each sex (three WT and three* sfx *mice). The extraction and purification procedure were previously reported [12]. A total of 200 ng of high-quality RNA was used to generate cDNA and cRNA by using an Affymetrix GeneChip system with genome 430 2.0 arrays. After *P*value assessment (*P* < 0.05) [9, 10] and filtering for fold changes ≤2 [13], differentially expressed genes were then used for comparison and analysis. Genes were notated as “present,” “absent,” or “May be present” according to Gatti et al. [13].

### 2.3. Whole Genome Expression Profiles of Female and Male Mice of BXD RI Strains

Data for this study come from gene expression profiles using whole genome expression data from spleens of male mice (http://www.genenetwork.org/webqtl/main.py?FormID=sharinginfo&GN_AccessionId=286) and female mice BXD strains (http://www.genenetwork.org/webqtl/main.py?FormID=sharinginfo&GN_AccessionId=287). The data include gene expression profiles of a total of 81 strains. Two arrays were processed per strain, one for males and one for females. The sex matched strains included two progenitors (B6, D2), An F1, 63 BXD strains (BXD1, BXD2, BXD5, BXD6, BXD8, BXD9, BXD11, BXD14, BXD15, BXD16, BXD18, BXD19, BXD20, BXD21, BXD24, BXD25, BXD27,B XD28, BXD31, BXD32, BXD33, BXD38, BXD39, BXD40, BXD42, BXD44, BXD45, BXD49, BXD51, BXD55, BXD56, BXD60, BXD63, BXD64, BXD65, BXD66, BXD67, BXD68, BXD69, BXD70, BXD71, BXD73, BXD74, BXD75, BXD77, BXD78, BXD79, BXD81, BXD83, BXD84, BXD85, BXD86, BXD87, BXD90, BXD92, BXD95, BXD96, BXD97, BXD98, BXD99, BXD101, BXD102, and BXD103), and 18 common strains.

### 2.4. Association of Expression Levels among Genes

In order to elucidate the molecular pathways among genes differentially expressed between female and male* sfx *mice, we examined the associations among those genes using gene expression profiles of spleen in BXD RI strains. The spleen of untreated young adult mice was profiled using the Affymetrix GeneChip Mouse Gene 1.0 ST array at Dr. Williams' laboratory. Mouse Gene 1.0 ST contains approximately 34,728 probe sets that target approximately 29,000 well-defined transcripts (RefSeq mRNA isoforms) and essentially all known protein coding genes in mouse. Expression values were logged and then were further normalized and rescaled so that the mean value for each array data set is 8 units with a standard deviation of 2 units. The whole genome expression data of spleens has been posted on the GeneNetwork webpage at http://www.genenetwork.org/webqtl/main.py. In case of multiple probes of a gene, the probe with the highest expression level was chosen to represent the gene. Identification of bone relevant genes was conducted with a searching tool, PGMapper (http://www.genediscovery.org/pgmapper/index.jsp) with Key word “bone.” For any potential candidates, at least the abstract of one reference was read by two authors to determine a link between the gene and bone. For a gene with more than one reference that indicated its candidacy, at least two references were read and cited in this study.

## 3. Results 

### 3.1. Affymetric Data Quality Checking

Quality checking indicated that the whole gene expression profile generated from Affymetrix platform in our laboratory is reliable. Previously, we have used real time RT-PCR or semiquantitative RT-PCR to confirm our data from microarray [9]. In the past 10 years, we have processed thousands of Affymetrix gene expression arrays. Recently, evidence has shown that microarray is reliable because of the newly development of technology and analytical tool [13, 14]. We carefully checked the quality of the microarray data before we utilized them. To increase the accuracy of signal and fold change calculations, the probe logarithmic intensity error estimation (PLIER) algorithm method was applied to generate the intensity values of transcripts together with the MAS5 method for generating the detection calls (present, marginal, or absent) using Expression Console software (Affymetrix). After detection call (absent) and fold change (≤2.0) filtering, the significance of gene expression changes between wild type and* sfx* mice (expressed as a *q* value) was determined using EDGE software, which proved to be superior to current leading methods [13, 15]. In addition, we have confirmed the expression of several oxidative genes by real time RT-PCR in an analysis of gender differences of oxidative pathway using the same sets of microarray data. Therefore, the data for this study is accurate and reliable.

### 3.2. Differential Gene Expression Levels in Female and Male in* sfx* and Wt Controls

**Figure 1:**
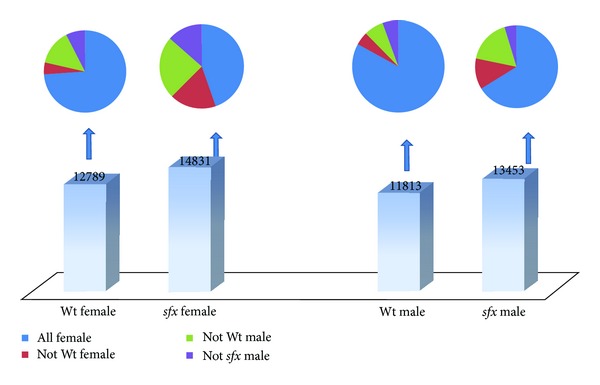
Comparison of whole genome gene expression levels in female and male in* sfx *and Wt controls. Bottom of the figure indicate the number of genes that are notated as “present” according to their expression level in each four categories (*sfx *Female,* sfx *Male, Wt Female, and Wt Male). Not all genes presented in one category will be “present” in other categories. The up portion of the figure indicates the percentages of “present” or “absent” of genes in four types of mice, all of which are “present” in each category below in the bottom portion of the figure.

We examined the expression levels of whole genome level between male and female. We first looked at how many genes are expressed based on the Affymetrix criteria. According to the microarray measurements, the percentages of transcripts called “presence” or “maybe presence” in the normal femur in female and male mice are 56.36% (12789/22690), and 52.06% (11813/22690) and in* sfx *mice are 65.36% (14831/22690) and 59.29% (13453/22690), respectively (Figure 1). Thus, it appears that more transcripts were expressed at “present” level in* sfx *mice in both sexes than that of the Wt control. On the other hand, more transcripts expressed at higher level in male than that in female in both* sfx *and Wt controls. While the majority of probes present in male when they present in female, many probes present in either male or female only.

We found that, in general, female mice in both wild type and* sfx* mice have more genes at detectable level than that in male mice. In the Wt female mice, among 12789 transcripts categorized as “presence” or “maybe presence,” 597 of those transcripts were categorized as “absent” in* sfx *female mice, 1780 in male Wt mice and 960 in* sfx *male mice (Figure 1). In the Wt male mice, among 11813 transcripts categorized as “presence” or “maybe presence”, 657 of those transcripts were categorized as “absent” in* sfx *male mice, 804 in female Wt mice and 546 in* sfx *female mice (Figure 1). In the* sfx *female mice, among 14831 transcripts categorized as “presence” or “maybe presence,” 2639 of those transcripts were categorized as “absent” in Wt female mice, 3564 in male Wt mice and 2004 in* sfx *male mice (Figure 1). In the* sfx *male mice, among 13453 transcripts categorized as “presence” or “maybe presence,” 2297 of those transcripts were categorized as “absent” in Wt male mice, 1624 in female Wt mice and 629 in* sfx *female mice (Figure 1).

### 3.3. Gene Expression Profiling in Female and Male Mice between* sfx* and Wt Mice

**Figure 2:**
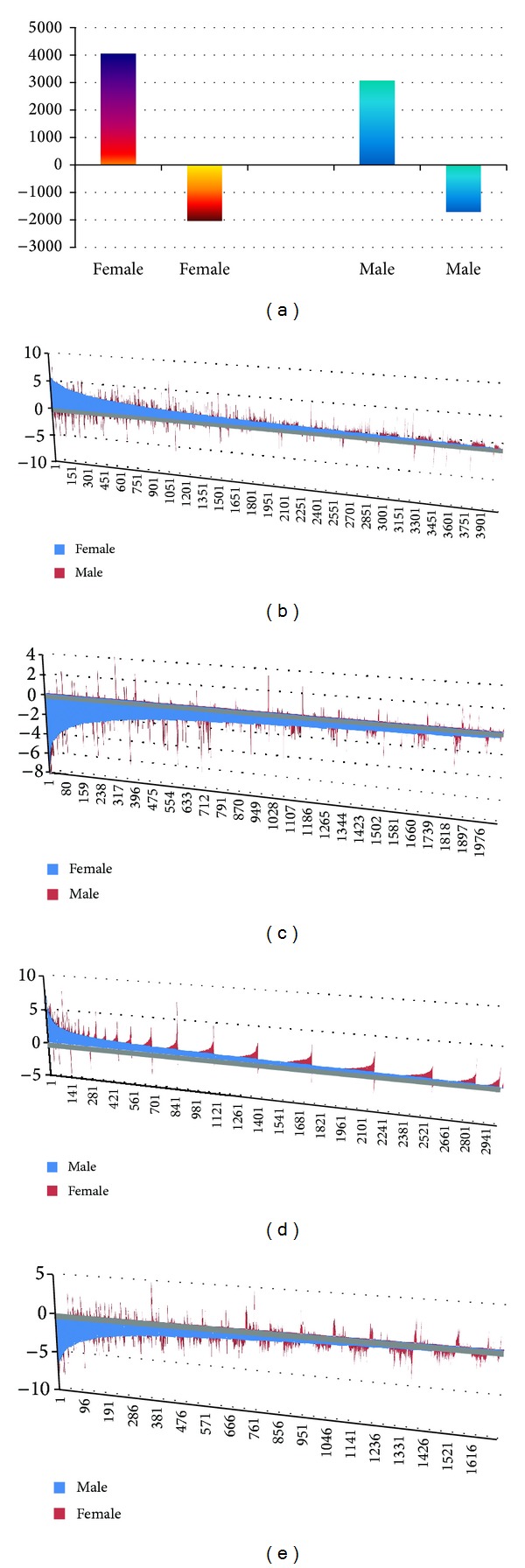
Genes are up- and downregulated in* sfx *mice. (a) shows the number of genes that are up- and downregulated by* Gulo* in female and male mice. *Y* axis in (b), (c), (d), and (e) indicates the fold changes of expression level in* sfx *mice. *X* axis indicates the order number of genes shown in each figure. (b) shows the upregulated genes in* sfx *female (shown in Blue color) and their expression level in male mice (In red color). (c) shows the downregulated genes in* sfx *female (shown in Blue color) and their expression level in male mice (in red color). (d) shows the upregulated genes in* sfx *male (shown in blue color) and their expression level in female mice (in red color). (e) shows the downregulated genes in* sfx *male (shown in blue color) and their expression level in female mice (in red color).

We then compared the expression profiles of genes in female and in male separately. We found more upregulated genes in female than that in male mice. In the female, 6082 transcripts were found to be differentially expressed in femurs between the* sfx *and Wt mice. The express levels of only 369 transcripts either in* sfx *or in Wt are categorized as “absent.” The expression levels of the rest of those differentially expressed transcripts are all categorized as “presence” or “maybe presence” in both* sfx *and Wt mice. Among 6082 transcripts, 2039 of those transcripts were downregulated in* sfx *mice while 4043 were upregulated in* sfx *mice (Figure 2(a)). Thus, the upregulated probes were two times more than that of downregulated in female* sfx *mice.

We next found 11 that many down regulated genes in female mice are not differentially expressed in the male mice. Among 2039 downregulated transcripts in female* sfx *mice, 50 are upregulated in male, 1114 were categorized as none change (Figure 2(b)). Thus, a total of 845 probes are commonly downregulated between male and female mice. Among 4043 upregulated probes in* sfx *female mice, 104 were downregulated in male, 2058 were categorized as none change. Thus, 1881 upregulated probes in female are also upregulated or possibly upregulated in male. Thus, more than half of those down- and upregulated probes in female* sfx *mice were not down- or upregulated in male* sfx *mice (Figure 2(c)).

We also found in male mice more differentially regulated genes in* sfx* mice than that in wild type mice. In the male, 4772 transcripts were found to be differentially expressed in femurs between* sfx *and Wt mice. The express levels of 405 transcripts either in* sfx *or in Wt are categorized as “absent,” while the rest are all categorized as “presence” or “maybe presence” in both* sfx *and Wt mice. Among 4772 transcripts, 1702 of those transcripts were downregulated in* sfx *mice while 3070 were upregulated in* sfx *mice (Figure 2(a)). Similar to that of female mice, much more probes are upregulated than downregulated probes in* sfx *mice.

We found that majority expressed and downregulated genes in male wild type mice are also down regulated in female wild type mice. Among 1702 downregulated transcripts in male Wt mice, 753 were categorized as none change (Figure 2(d)). Thus, 104 are upregulated in female and 845 are commonly downregulated between male and female. Among 3070 upregulated probes in Wt male mice, 50 were down regulated in female, 1146 were categorized as none change (Figure 2(e)). A total of 1873 probes are upregulated in female are upregulated or possibly upregulated in male mice. Thus, more than third of those down- and upregulated probes in male Wt mice were not down- or upregulated in female Wt mice.

The sex differentially expressed genes include a large number of known bone relevant genes. All together, we obtained a total of 154 sex specific expressed probes, or sex opposite expression probes, including the 50 probes downregulated in female while they are upregulated in male (See Supplementary Material available online at http://dx.doi.org/10.1155/2014/584910; Table s1) and 104 probes are upregulated in female while down regulated in male (supplementary Table s2). The gene expressions of several well-known bone formation genes including* Msx2*,* Pxr*,* Pou2f1*,* Cbfa1*,* Gata1*,* Bmp8*,* Sox9*,* Runx2*, and* Alp* [16–18] were not changed significantly between* sfx *and wild type control. The 50 probes represent 49 genes and ESTs. By searching bone relevant genes with PGMapper [19] using key word “bone,” we found 14 genes which are in the literatures with bone and the name of the gene in the same article. The 104 probes represent 98 genes and ESTs. By searching bone relevant genes with PGMapper [19] using key word “bone,” we found 40 genes which are in the literatures with bone and the name of the gene in the same article.

### 3.4. Gene Network of Differentially Expressed and Bone Relevant Genes in Spleen of Male Mice of BXD Strains

**Figure 3:**
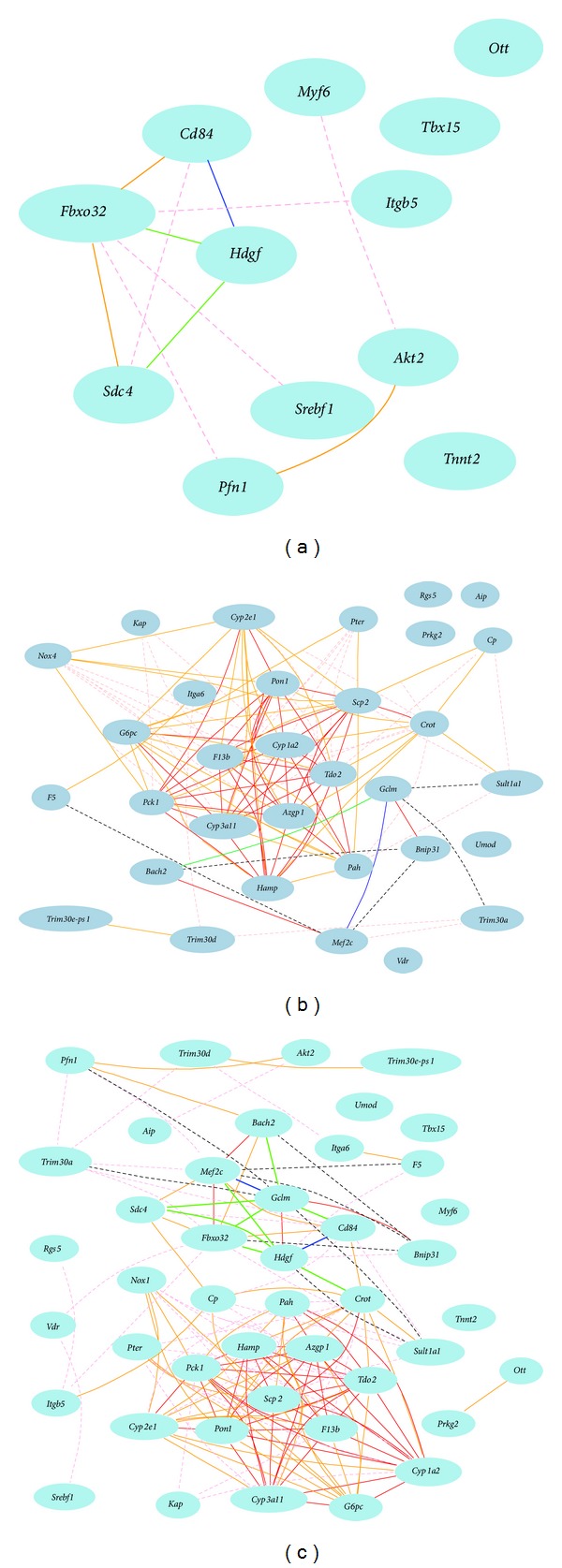
Gene network among bone relevant genes, which are upregulated in female while downregulated in male, constructed using expression profiles from spleen of male BXD mouse strains. (a) The 12 nodes in the graph below show the selected traits. All nodes are displayed. The 11 edges between the nodes, filtered from the 66 total edges and drawn as curves, show Pearson correlation coefficients greater than 0.35 or less than −0.35. The graph's canvas is 40.0 by 40.0 cm and the node labels are drawn with a 14.0 point font and the edge labels are drawn with a 14.0 point font. (b) The 32 nodes in the graph below show the selected traits. All nodes are displayed. The 117 edges between the nodes, filtered from the 496 total edges and drawn as curves, show Pearson correlation coefficients greater than 0.4 or less than −0.4. The graph's canvas is 40.0 by 40.0 cm and the node labels are drawn with a 14.0 point font and the edge labels are drawn with a 14.0 point font. (c) The 44 nodes in the graph below show the selected traits. All nodes are displayed. The 156 edges between the nodes, filtered from the 946 total edges and drawn as curves, show Pearson correlation coefficients greater than 0.4 or less than −0.4. The graph's canvas is 40.0 by 40.0 cm and the node labels are drawn with a 14.0 point font and the edge labels are drawn with a 10.0 point font.

We first examined the Gene Network of 14 bone relevant genes which are downregulated in female and upregulated in male in* sfx* mice using the gene expression profiles of spleen from male BXD mice. We obtained probes for 12 genes of these 12 genes. Gene network construction indicated that in male mice expression level of hepatoma-derived growth factor (*Hdgf*) has a strong negative association with that of* Cd84* and a reasonable negative correlation with that of syndecan 4 (*Sdc4*) and F-box only protein 32 (*Fbxo32*), while* Fbxo32* and* Sdc4* are positively associated each other. It has been known that* Sdc4* supports bone fracture repair [12]. Its association to the* Fbxo32*, a gene known for skeletal muscle atrophy, suggests a potentially important role of both genes in bone pathology.* Hdgf* has been regarded as dispensable for normal mouse development but its expression is associated with common tumors of bone in children and young adults. Its negative correlation with* Cd84*, a marker for B cells, suggests a potentially important function in immune system of* Hdgf, *most likely in bone marrow. The rest of the genes have weak negative or positive correlations (Figure 3(a)).

We next examined the 40 bone relevant genes which are upregulated in female and downregulated in* sfx *male. We obtained 32 probes for 30 genes from gene profiles of spleen of male mice of BXD strains. We found that, unlike genes that are downregulated in female and upregulated in male, majority of these genes are positively correlated while half of these probes have strong positive correlations (Figure 3(b)). Among them, there is a cluster of genes strong positively correlated to each other. Genes in this cluster are* Azgp1*,* Pck1*,* Cyp2e1*,* Pon1*,* Hamp*,* Cyp1a2*,* F13b*,* Tdo2*,* Scp2*,* G6pc*,* Cyp3a11*,* Pah*, and* Crot*. Members of  P450 family genes,* Cyp1a2*,* Cyp2e1*,* Cyp3a11*, form the core of the positive network. In humans, polymorphisms of many P450 family genes have been found to be linked to bone density. Very importantly, CYP1A2 polymorphisms have been linked to bone mineral density of the proximal femur in elderly men but not in women [20]. In our analysis,* Cyp1a2* is strongly directly positively correlated to 10 genes in this 13 gene cluster (Figures 3(b) and 3(c)). The link among members of P450 family genes and others is the first and the critic clue for their potential function in sex differential development and pathology. The only pair of negative association is between the* Gclm* and* Metf2c*, which in turn is positively associated with* Bach2*.

We then combined all 12 and 32 bone relevant up- and downregulated genes using gene expression profiles of spleen of male mice. The gene network demonstrates that there are two cluster centers in associations of those genes (Figure 3(c)). One is the major positive cluster which is the same as that in 32 probes. The other is the new cluster of mixture of positive and negative associations. This cluster is formed by a new connection between* Gclm* in 32 probe group and* Hdgf *in the 12 probe group. The association between* Hdgf *and* Gclm, *a glutamate-cysteine ligase previously known connected to myocardial infarction [21], reveals novel function of* Gclm* as well as the new molecular pathway of these two genes in bone.

### 3.5. Gene Network of Differentially Expressed and Bone Relevant Genes in Spleen of Female Mice of BXD Strains

**Figure 4:**
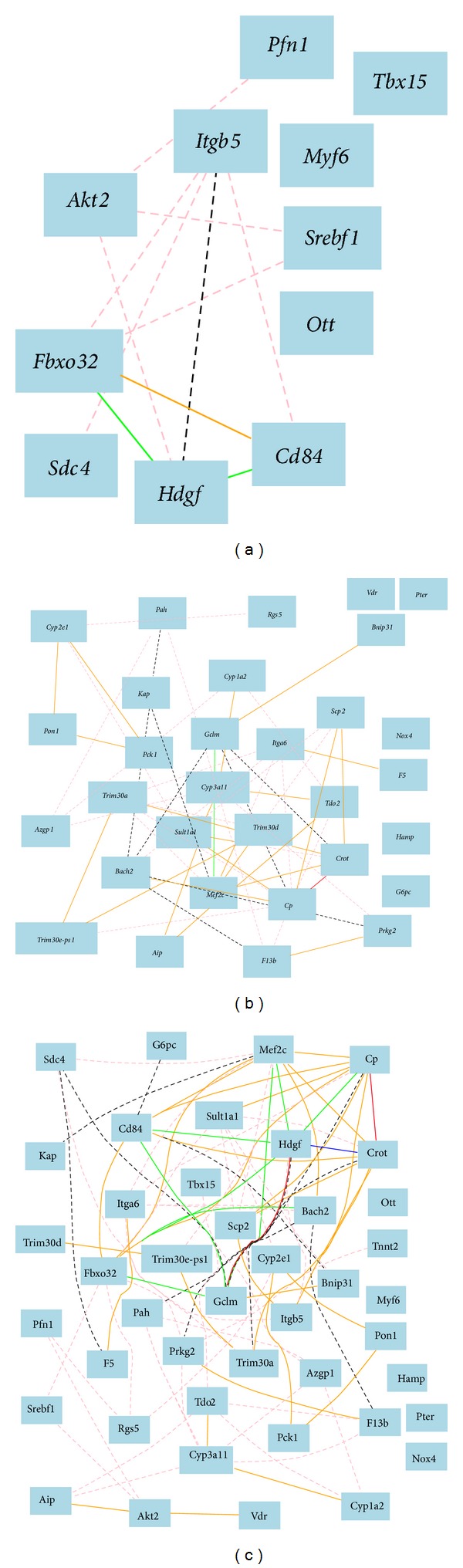
Gene network among bone relevant genes, which are upregulated in female while downregulated in female, constructed using expression profiles from spleen of female BXD mouse strains. (a) The 11 nodes in the graph below show the selected traits. All nodes are displayed. The 11 edges between the nodes, filtered from the 55 total edges and drawn as curves, show Pearson correlation coefficients greater than 0.35 or less than −0.35. The graph's canvas is 40.0 by 40.0 cm and the node labels are drawn with a 14.0 point font and the edge labels are drawn with a 10.0 point font. (b) The 31 nodes in the graph below show the selected traits. All nodes are displayed. The 49 edges between the nodes, filtered from the 465 total edges and drawn as curves, show Pearson correlation coefficients greater than 0.4 or less than −0.4. The graph's canvas is 40.0 by 40.0 cm and the node labels are drawn with a 14.0 point font and the edge labels are drawn with a 14.0 point font. (c) The 43 nodes in the graph below show the selected traits. All nodes are displayed. The 88 edges between the nodes, filtered from the 903 total edges and drawn as curves, show Pearson correlation coefficients greater than 0.4 or less than −0.4. The graph's canvas is 40.0 by 40.0 cm and the node labels are drawn with a 14.0 point font and the edge labels are drawn with a 14.0 point font.

To investigate whether the gene network in female mice is the same or similar to that of male mice, we conducted the similar analysis of gene network of three groups of genes in spleens of female BXD strains. We first examined the gene network of 14 bone relevant genes downregulated in female while they are upregulated in male in gene expression profiles of female mice. Similarly, we obtained 12 probes represent those 12 genes from the female spleen gene profiles (Figure 4(a)). Unlike in male mice, gene network analysis indicates that there is no strong correlation among these genes.* Sdc4* shows no association with* Hdgf* and* Fbxo32*.

We next examined the 40 bone relevant genes upregulated in female while downregulated in female (Figure 4(b)). We obtained 31 probes for 29 genes from gene profiles of spleen of female mice. There is no clear cluster of associated genes. There is only one pair of strong negative association between ceruloplasmin (*Cp*) and carnitine octanoyltransferase (*Crot*).

We finally analyzed the all 12 and 31 probes of bone relevant up- and downregulated genes using gene expression profiles of spleen of female mice (Figure 4(c)). Gene network shows no positive cluster. There is only one group of mixture connected genes among* Gclm*,* Hdgf*,* Crot*, and* Cp*. Overall, the gene network of these selected genes in female mice is largely different from that of the male mice.

### 3.6. Potential Function of Differential Expressed Genes in Collagen Synthesis and Metabolism

We examined the potential function connection between the sex differential expressed genes and the collagen synthesis. VC is well known to regulate the collagen synthesis [22, 23]. It is reasonable to believe that at least some of genes in these up- and downregulated genes in* sfx* mice are directly or indirectly connected to collagen synthesis. We therefore conducted a research for these genes in PGMapper with “collagen” as the key word. The output from the search indicates that many of these genes are connected to collagen. From the genes that are upregulated in female and downregulated in male mice, we found that more than 20 genes (*Cp*,* Vdr*,* Pah*,* Nox4*,* Cyp2e1*,* Cyp1a2*,* Mef2c*,* F5*,* Pon1*,* Itga6*,* Umod*,* Pso*,* Umod*,* Rgs5*,* Hamp*,* Pter*,* Cyp2c29*,* Cyp3a11*,* Aip-1*,* Bhmt*,* Fbp1*,* Prkg2*,* Sult1a1*,* Scp2*,* Slc10a1*,* G6pc*,* Ass1*, and* Kap*) are connected to collagen. Particularly, several important genes have been known to regulate the collagen synthesis and metabolism including ceruloplasmin (*Cp*) vitamin D receptor (*Vdr*), phenylalanine hydroxylase (*Pah*), NADPH oxidase 4 (*Nox4*), cytochrome P450, 2e1 (*Cyp2e1*),* Cyp1a2*,* Cyp2c29*,* Cyp3a11*, MADS box transcription enhancer factor 2, polypeptide C (*Mef2c*), coagulation factor V (F*5*), paraoxonase 1 (*Pon1*), and integrin alpha 6 (*Itga6*). From genes that are downregulated in female and upregulated in male* sfx* mice, we found 13 genes (*Akt2*,* Sdc4*,* Myf6*,* Pabpn1*,* Itgb5*,* Tnnt2*,* MAP1*,* Pfn1*,* Tsix*,* Xist*,* Srebf1*,* Arf5*, and* Hdgf) *that are connected to collagen synthesis. This data also suggests the importance of collagen differential production in regulation of skeletal structures between male and female.

## 4. Discussion

By analyzing the female and male mice separately, we found many more differential expressed genes between wild type and* sfx *mice from either female or male mice than we found previously using RNA of mixture of female and male mice [9, 10]. It is obvious that the skeletal system is different between female and male. The female and male skeletal system is most likely to react the VC deficiency in* sfx *mice differently. The comparison between disease and control samples using data of sex mixture suffers from neutralization of gene expression levels between female and male. This result suggests that in the study of genes that are potentially affected by the sex or the gender, data from female and male individuals should be analyzed separately. Many reports have been using the sex balanced data of mouse strains. Our data poised a question of whether the balanced data should be used without understanding of the sex differences.

In* sfx *mice, we used the data from femurs. In the BXD RI strains, we used the data from the spleen rather than the bone, because of the available of gene expression profiles from spleens of both sexes of those mice. Although there are differences between those two sets of data, their conclusions are compatible considering the degree of similarity on the sex differences between the data from the spleen of BXD mice and the femur of* sfx *mice. While the sex difference in gene pathways is clearly demonstrated in our study, the exact genes in those pathways in mice may be or may not be the same to that in humans. Along with the deletion of* Gulo* gene in human genome, other genes are also deleted or the pathways have been altered. For example, urate oxidase gene (*Uox*) is active in most mammals. The activity of* Uox* catalyzes the oxidation of uric acid to allantoin; however, humans and some primates lack this enzyme activity. During the evolution, human body has developed pathways in adopt of the loss of Gulo gene, human body has developed pathways in adopt such a loss. However, VC like other vitamins is essential to human skeletal development. The same is to the mouse skeletal system. As such, at least some of those key genes identified from this study in certain degree are applicable to humans.

One of the critical finding of the study is the cluster of male specific strong positively associated genes. Three P450 genes,* Cyp1a2*,* Cyp2e1*,* Cyp3a11*, form the core of the positive cluster. Recently, several publications have reported the study on polymorphisms and bone mineral density in human populations. Hallström and colleagues reported that genotypes for cytochrome P450 1A2 (CYP1A2) associated with metabolism of caffeine and bone mineral density in men [20]. Hong et al. reported that genetic variations in the CYP19A1 gene are significantly associated with BMD at different skeletal sites in adult men, but not in women [24]. Yamada et al. reported that CYP17A1 and MTP are susceptibility loci for increased BMD in postmenopausal and premenopausal Japanese women, respectively, and that VLDLR constitutes such a locus in Japanese men [25]. Considering the fact that polymorphisms of many genes in P450 have been linked to bone mineral density in human populations in a gender specific manner, further study using mouse model may bring essential information on the molecular mechanism of the sex specific regulation of mineral bone density by P450 family and their associated genes.

In previously microarray based studies, we conducted real time PCR to confirm the data from microarray [9]. As microarray technology has been developed for more than a decade, and the analytic methodology has been mature, we did not conduct the real time PCR for the verification of the bone relevant genes of microarray data from the* sfx *mice and their control mice. However, we have confirmed the differential expression levels of a list of oxidative genes from the same set of microarray data by real time RT PCR [26]. In addition, similarity of the result of data from spleen of BXD mice and the* sfx *mice convinced us that those data are relievable. Plus, multiple probes from several genes showed similar result, and again confirmed the reliability of the data.

In this study, we analyzed the whole genome expression profiles of* sfx* and its wild type at one time point. The sex and age dependent variation of bone structure has been investigated by Willinghamm et al. [27] and Marano et al. [28]. The differences on bone development and structure between* sfx* and its wild type mice, Balb/c, have been reported previously [7, 8]. Based on the information that mouse bone develops and matures at 4 month of age [29], we believe that the gene expression profile at 6 weeks of age represents the best stage of gene expression level of mouse bone. At the 6 weeks, the bone grows rapidly but not entering mature stage [29]. Therefore, activities of essential genes for bone growth and structuring are high. While in mature stage of bone, we may not be able to detect the differential activities of many bone genes. However, gene expression levels may vary at different time points [26, 27]. Future study for the effect of VC on the gene expression profile and structure changes at different time points will enhance our understanding of bone regulation by VC.

## Supplementary Material

Supplementary Table s1: The able contains information of the genes that are up regulated in female and down regulated in male sfx mice. P=presence; A= absent; D= decrease (of expression level); I= increase (of expression level).
Supplementary Table s2: The table contains information of genes that are up regulated in male and down regulated in female sfx mice. P=presence; A= absent; D= decrease (of expression level); I= increase (of expression level).

## References

[1] Levine M, Wang Y, Padayatty SJ, Morrow J (2001). A new recommended dietary allowance of vitamin C for healthy young women. *Proceedings of the National Academy of Sciences of the United States of America*.

[2] Fain O, Pariés J, Jacquart B (2003). Hypovitaminosis C in hospitalized patients. *European Journal of Internal Medicine*.

[3] Maruyama C, Araki R, Takeuchi M (2004). Relationships of nutrient intake and lifestyle-related factors to serum folate and
plasma homocysteine concentrations in 30–69 year-old Japanese. *Journal of Nutritional Science and Vitaminology*.

[4] Geber J, Murphy E (2012). Scurvy in the great irish famine: Evidence of vitamin C deficiency from a mid-19th century skeletal population. *American Journal of Physical Anthropology*.

[5] Panda AK, Ruth RP, Padhi SN (1984). Effect of age and sex on the ascorbic acid content of kidney, skeletal muscle and pancreas of common Indian toad, Bufo melanostictus. *Experimental Gerontology*.

[6] Kuo SM, MacLean ME, McCormick K, Wilson JX (2004). Gender and sodium-ascorbate transporter isoforms determine ascorbate concentrations in mice. *Journal of Nutrition*.

[7] Beamer WG, Rosen CJ, Bronson RT (2000). Spontaneous fracture (*sfx*): a mouse genetic model of defective peripubertal bone formation. *Bone*.

[8] Jiao Y, Li X, Beamer WG (2005). A deletion causing spontaneous fracture identified from a candidate region of mouse chromosome 14. *Mammalian Genome*.

[9] Yan J, Jiao Y, Li X (2007). Evaluation of gene expression profiling in a mouse model of L-gulonolactone oxidase gene deficiency. *Genetics and Molecular Biology*.

[10] Jiao Y, Zhang J, Yan J (2011). Differential gene expression between wild-type and Gulo-deficient mice supplied with vitamin C. *Genetics and Molecular Biology*.

[11] Andreux PA, Williams EG, Koutnikova H (2012). Systems genetics of metabolism: the use of the BXD murine reference panel for multiscalar integration of traits. *Cell*.

[12] Bertrand J, Stange R, Hidding H (2013). Syndecan 4 supports bone fracture repair, but not fetal skeletal development, in mice. *Arthritis and Rheumatism*.

[13] Gatti D, Maki A, Chesler EJ (2007). Genome-level analysis of genetic regulation of liver gene expression networks. *Hepatology*.

[14] Unuma K, Shintani-Ishida K, Yahagi N (2010). Restraint stress induces connexin-43 translocation via *α*-adrenoceptors in rat heart. *Circulation Journal*.

[15] Archer KJ, Reese SE (2009). Detection call algorithms for high-throughput gene expression microarray data. *Briefings in Bioinformatics*.

[16] Xiao Y, Cui J, Li YX, Shi YH, Le GW (2010). Expression of genes associated with bone resorption is increased and bone
formation is decreased in mice fed a high-fat diet. *Lipids*.

[17] Lee D, Yeh C, Chang S (2008). Integrin-mediated expression of bone formation-related genes in osteoblast-like cells in response to fluid shear stress: roles of extracellular matrix, Shc, and mitogen-activated protein kinase. *Journal of Bone and Mineral Research*.

[18] Helvering LM, Liu R, Kulkarni NH (2005). Expression profiling of rat femur revealed suppression of bone formation genes by treatment with alendronate and estrogen but not raloxifene. *Molecular Pharmacology*.

[19] Xiong Q, Qiu Y, Gu W (2008). PGMapper: a web-based tool linking phenotype to genes. *Bioinformatics*.

[20] Hallström H, Melhus H, Glynn A, Lind L, Syvänen AC, Michaëlsson K (2010). Coffee consumption and CYP1A2 genotype in relation to bone mineral density of the proximal femur in elderly men and women: a cohort study. *Nutrition & Metabolism*.

[21] Nakamura S, Kugiyama K, Sugiyama S (2002). Polymorphism in the 5′-flanking region of human glutamate-cysteine ligase modifier subunit gene is associated with myocardial infarction. *Circulation*.

[22] Barnes MJ (1975). Function of ascorbic acid in collagen metabolism. *Annals of the New York Academy of Sciences*.

[23] Vogel Z, Daniels MP, Chen T (1987). Ascorbate-like factor from embryonic brain. Role in collagen formation, basement membrane deposition, and acetylcholine receptor aggregation by muscle cells. *Annals of the New York Academy of Sciences*.

[24] Hong X, Hsu Y, Terwedow H (2007). CYP19A1 polymorphisms are associated with bone mineral density in Chinese men. *Human Genetics*.

[25] Yamada Y, Ando F, Shimokata H (2005). Association of polymorphisms in CYP17A1, MTP, and VLDLR with bone mineral density in community-dwelling Japanese women and men. *Genomics*.

[26] Jiao Y, Chen H, Yan J (2013). Genome-wide gene expression profiles in antioxidant pathways and their potential sex differences and connections to Vitamin C in mice. *International Journal of Molecular Sciences*.

[27] Willinghamm MD, Brodt MD, Lee KL, Stephens AL, Ye J, Silva MJ (2010). Age-related changes in bone structure and strength in female and male BALB/c Mice. *Calcified Tissue International*.

[28] Marano RJ, Tickner J, Redmond SL (2013). Prolactin expression in the cochlea of aged BALB/c mice Is gender biased and correlates to loss of bone mineral density and hearing loss. *PLoS ONE*.

[29] Beamer WG, Donahue LR, Rosen CJ, Baylink DJ (1996). Genetic variability in adult bone density among inbred strains of mice. *Bone*.

