# Supplementary material for: Genome Wide Analysis of Sex Difference in Gene Expression Profiles of Bone Formations Using sfx Mice and BXD RI Strains
Source: ScientificWorldJournal. 2014 Jul 14;2014:584910. doi: 10.1155/2014/584910 (PMC4123512; doi:10.1155/2014/584910)
Supplement: Supplementary file 1 — Supplementary Table s1: The able contains information of the genes that are up regulated in female and down regulated in male sfx mice. P=presence; A= absent; D= decrease (of expression level); I= increase (of expression level). Supplementary Table s2: The table contains information of genes that are up regulated in male and down regulated in female sfx mice. P=presence; A= absent; D= decrease (of expression level); I= increase (of expression level). [file 584910.f1.docx]

Supplementary Table S1. Genes up regulated in female and down regulated in male sfx mice

| gene aff id | Balb-F-bone_Signal | Balb-F-bone_Detection | Sfx-F-bone_Signal | Balb-F-bone_Signal Log Ratio | Balb-F-bone_Change | Sfx-F-bone_Detection | Balb-M-bone_Signal | Balb-M-bone_Detection | Sfx-M-bone_Signal | Sfx-M-bone_Detection | Sfx-M-bone_Signal Log Ratio | Sfx-M-bone_Change | Descriptions | Detailed Description |
| --- | --- | --- | --- | --- | --- | --- | --- | --- | --- | --- | --- | --- | --- | --- |
| 1415968_a_at | 466.3 | P | 555.3 | 0.4 | I | P | 11904.5 | P | 1130 | P | -3.3 | D | Kap | gb:NM_010594.1 /DB_XREF=gi:6754405 /GEN=Kap /FEA=FLmRNA /CNT=383 /TID=Mm.13052.1 /TIER=FL+Stack /STK=155 /UG=Mm.13052 /LL=16483 /DEF=Mus musculus kidney androgen regulated protein (Kap), mRNA. /PROD=kidney androgen regulated protein /FL=gb:BC013453.1 gb:NM_010594.1 gb:M22810.1 |
| 1415994_at | 5048.3 | P | 8964.2 | 0.8 | I | P | 6411.7 | P | 2329 | P | -1.5 | D | Cyp2e1 | gb:NM_021282.1 /DB_XREF=gi:11276064 /GEN=Cyp2e1 /FEA=FLmRNA /CNT=381 /TID=Mm.21758.1 /TIER=FL+Stack /STK=151 /UG=Mm.21758 /LL=13106 /DEF=Mus musculus cytochrome P450, 2e1, ethanol inducible (Cyp2e1), mRNA. /PROD=cytochrome P450, 2e1, ethanol inducible /FL=gb:NM_021282.1 gb:L11650.1 gb:BC013451.1 |
| 1416129_at | 1754.5 | P | 2657.5 | 0.6 | I | P | 4172.8 | P | 1885.7 | P | -1.3 | D | 1300002F13Rik | gb:NM_133753.1 /DB_XREF=gi:19526961 /GEN=1300002F13Rik /FEA=FLmRNA /CNT=270 /TID=Mm.21679.1 /TIER=FL+Stack /STK=123 /UG=Mm.21679 /LL=74155 /DEF=Mus musculus RIKEN cDNA 1300002F13 gene (1300002F13Rik), mRNA. /PROD=RIKEN cDNA 1300002F13 /FL=gb:BC005546.1 gb:NM_133753.1 |
| 1416239_at | 335.8 | P | 2456.8 | 2.9 | I | P | 1625.5 | P | 501.1 | P | -1.8 | D | Ass1 | gb:NM_007494.1 /DB_XREF=gi:6996910 /GEN=Ass1 /FEA=FLmRNA /CNT=282 /TID=Mm.3217.1 /TIER=FL+Stack /STK=108 /UG=Mm.3217 /LL=11898 /DEF=Mus musculus argininosuccinate synthetase 1 (Ass1), mRNA. /PROD=arginosuccinate synthetase 1 /FL=gb:M31690.1 gb:NM_007494.1 gb:BC002074.1 |
| 1416779_at | 199 | P | 309.5 | 0.7 | I | P | 434.4 | P | 120.9 | P | -1.8 | D | Sdpr | gb:BE197945 /DB_XREF=gi:8710114 /DB_XREF=ug76h12.x1 /CLONE=IMAGE:1548359 /FEA=FLmRNA /CNT=150 /TID=Mm.220962.1 /TIER=Stack /STK=10 /UG=Mm.220962 /LL=20324 /UG_GENE=Sdpr /UG_TITLE=serum deprivation response /FL=gb:NM_138741.1 gb:BC027005.1 gb:BC020008.1 |
| 1416809_at | 3235.6 | P | 7783.5 | 1.2 | I | P | 4038 | P | 1987.5 | P | -0.9 | D | Cyp3a11 | gb:BC010528.1 /DB_XREF=gi:14714762 /FEA=FLmRNA /CNT=276 /TID=Mm.21193.1 /TIER=FL+Stack /STK=68 /UG=Mm.21193 /LL=13112 /UG_GENE=Cyp3a11 /DEF=Mus musculus, cytochrome P450, steroid inducible 3a11, clone MGC:18636 IMAGE:4194909, mRNA, complete cds. /PROD=cytochrome P450, steroid inducible 3a11 /FL=gb:NM_007818.1 gb:BC010528.1 |
| 1416980_at | 311.4 | P | 487.3 | 0.1 | I | P | 464 | P | 205.5 | P | -1.5 | D | 0610006F02Rik | gb:NM_027853.1 /DB_XREF=gi:13386263 /GEN=0610006F02Rik /FEA=FLmRNA /CNT=176 /TID=Mm.19281.1 /TIER=FL+Stack /STK=61 /UG=Mm.19281 /LL=71664 /DEF=Mus musculus RIKEN cDNA 0610006F02 gene (0610006F02Rik), mRNA. /PROD=RIKEN cDNA 0610006F02 /FL=gb:NM_027853.1 gb:BC024898.1 |
| 1417110_at | 207.8 | P | 358.3 | 0.7 | I | P | 379.9 | P | 273.1 | P | -0.7 | D | Man1a | gb:NM_008548.1 /DB_XREF=gi:6678787 /GEN=Man1a /FEA=FLmRNA /CNT=197 /TID=Mm.117294.1 /TIER=FL+Stack /STK=56 /UG=Mm.117294 /LL=17155 /DEF=Mus musculus mannosidase 1, alpha (Man1a), mRNA. /PROD=mannosidase 1, alpha /FL=gb:BC015265.1 gb:U04299.1 gb:NM_008548.1 |
| 1417210_at | 17 | A | 149.1 | 2.7 | I | P | 711.3 | P | 438.4 | P | -1.1 | D | Eif2s3y | gb:NM_012011.1 /DB_XREF=gi:7242147 /GEN=Eif2s3y /FEA=FLmRNA /CNT=84 /TID=Mm.20831.1 /TIER=FL+Stack /STK=53 /UG=Mm.20831 /LL=26908 /DEF=Mus musculus eukaryotic translation initiation factor 2, subunit 3, structural gene Y-linked (Eif2s3y), mRNA. /PROD=eukaryotic translation initiation factor 2,subunit 3, structural gene Y-linked /FL=gb:NM_012011.1 |
| 1417314_at | 212.5 | P | 391.6 | 1 | I | P | 313 | P | 205 | P | -0.8 | D | H2-Bf | gb:NM_008198.1 /DB_XREF=gi:6996918 /GEN=H2-Bf /FEA=FLmRNA /CNT=187 /TID=Mm.653.1 /TIER=FL+Stack /STK=48 /UG=Mm.653 /LL=14962 /DEF=Mus musculus histocompatibility 2, complement component factor B (H2-Bf), mRNA. /PROD=histocompatibility 2, complement componentfactor B /FL=gb:NM_008198.1 gb:BC005451.1 gb:M57890.1 |
| 1417494_a_at | 1297.1 | P | 3051.3 | 0.9 | I | P | 3646.5 | P | 1622.7 | P | -1.2 | D | Cp | gb:NM_007752.1 /DB_XREF=gi:6680996 /GEN=Cp /FEA=FLmRNA /CNT=332 /TID=Mm.13787.1 /TIER=FL+Stack /STK=43 /UG=Mm.13787 /LL=12870 /DEF=Mus musculus ceruloplasmin (Cp), mRNA. /PROD=ceruloplasmin /FL=gb:U49430.1 gb:NM_007752.1 |
| 1417495_x_at | 369.9 | P | 1582.7 | 1.8 | I | P | 1053 | P | 417.9 | P | -1 | D | Cp | gb:NM_007752.1 /DB_XREF=gi:6680996 /GEN=Cp /FEA=FLmRNA /CNT=332 /TID=Mm.13787.1 /TIER=FL+Stack /STK=43 /UG=Mm.13787 /LL=12870 /DEF=Mus musculus ceruloplasmin (Cp), mRNA. /PROD=ceruloplasmin /FL=gb:U49430.1 gb:NM_007752.1 |
| 1417496_at | 56.8 | A | 265.6 | 1.8 | I | P | 127.9 | P | 83.6 | P | -1 | D | Cp | gb:BB531328 /DB_XREF=gi:9583257 /DB_XREF=BB531328 /CLONE=E030016E11 /FEA=FLmRNA /CNT=332 /TID=Mm.13787.1 /TIER=Stack /STK=16 /UG=Mm.13787 /LL=12870 /UG_GENE=Cp /UG_TITLE=ceruloplasmin /FL=gb:U49430.1 gb:NM_007752.1 |
| 1417498_at | 319.4 | P | 481.9 | 0.6 | I | P | 418.3 | P | 184.9 | P | -0.7 | D | Serpinf2 | gb:NM_008878.1 /DB_XREF=gi:6679382 /GEN=Serpinf2 /FEA=FLmRNA /CNT=169 /TID=Mm.934.1 /TIER=FL+Stack /STK=43 /UG=Mm.934 /LL=18816 /DEF=Mus musculus serine (or cysteine) proteinase inhibitor, clade F (alpha-2 antiplasmin, pigment epithelium derived factor), member 2 (Serpinf2), mRNA. /PROD=plasmin inhibitor alpha 2 /FL=gb:NM_008878.1 gb:BC026756.1 |
| 1417531_at | 274.9 | A | 288.1 | 0.3 | I | P | 2379.1 | P | 199.3 | P | -3.6 | D | Cyp2j5 | gb:NM_010007.1 /DB_XREF=gi:6753585 /GEN=Cyp2j5 /FEA=FLmRNA /CNT=156 /TID=Mm.12838.1 /TIER=FL+Stack /STK=42 /UG=Mm.12838 /LL=13109 /DEF=Mus musculus cytochrome P450, 2j5 (Cyp2j5), mRNA. /PROD=cytochrome P450, 2j5 /FL=gb:U62294.1 gb:BC021624.1 gb:NM_010007.1 |
| 1417532_at | 77.3 | A | 127.6 | 0.7 | I | P | 666.3 | P | 150.5 | P | -2.2 | D | Cyp2j5 | gb:BF783609 /DB_XREF=gi:12088748 /DB_XREF=602109616F1 /CLONE=IMAGE:4238067 /FEA=FLmRNA /CNT=156 /TID=Mm.12838.1 /TIER=ConsEnd /STK=0 /UG=Mm.12838 /LL=13109 /UG_GENE=Cyp2j5 /UG_TITLE=cytochrome P450, 2j5 /FL=gb:U62294.1 gb:BC021624.1 gb:NM_010007.1 |
| 1417651_at | 1487 | P | 1869.7 | 0.7 | I | P | 1396.6 | P | 620 | P | -1.2 | D | Cyp2c29 | gb:NM_007815.1 /DB_XREF=gi:6681108 /GEN=Cyp2c29 /FEA=FLmRNA /CNT=164 /TID=Mm.20764.1 /TIER=FL+Stack /STK=39 /UG=Mm.20764 /LL=13095 /DEF=Mus musculus cytochrome P450, 2c29 (Cyp2c29), mRNA. /PROD=cytochrome P450, 2c29 /FL=gb:BC019908.1 gb:NM_007815.1 gb:D17674.1 gb:BC013895.1 |
| 1417667_a_at | 279.9 | P | 387.9 | 0.5 | I | P | 1063.3 | P | 480 | P | -1.4 | D | Pter | gb:NM_008961.1 /DB_XREF=gi:6679524 /GEN=Pter /FEA=FLmRNA /CNT=68 /TID=Mm.2008.1 /TIER=FL+Stack /STK=39 /UG=Mm.2008 /LL=19212 /DEF=Mus musculus phosphotriesterase related (Pter), mRNA. /PROD=phosphotriesterase related /FL=gb:BC003793.1 gb:U28016.1 gb:NM_008961.1 |
| 1417776_at | 765.7 | P | 1096.6 | 0.8 | I | P | 452.1 | P | 255.7 | P | -0.9 | D | Azgp1 | gb:NM_013478.1 /DB_XREF=gi:7304910 /GEN=Azgp1 /FEA=FLmRNA /CNT=98 /TID=Mm.30061.1 /TIER=FL+Stack /STK=36 /UG=Mm.30061 /LL=12007 /DEF=Mus musculus alpha-2-glycoprotein 1, zinc (Azgp1), mRNA. /PROD=alpha-2-glycoprotein 1, zinc /FL=gb:D21059.1 gb:NM_013478.1 |
| 1417880_at | 286.5 | P | 1124 | 1.8 | I | P | 2242.5 | P | 400.6 | P | -2.3 | D | G6pc | gb:NM_008061.1 /DB_XREF=gi:6679892 /GEN=G6pc /FEA=FLmRNA /CNT=82 /TID=Mm.18064.1 /TIER=FL+Stack /STK=34 /UG=Mm.18064 /LL=14377 /DEF=Mus musculus glucose-6-phosphatase, catalytic (G6pc), mRNA. /PROD=glucose-6-phosphatase, catalytic /FL=gb:BC013448.1 gb:U00445.1 gb:NM_008061.1 |
| 1417961_a_at | 73.8 | A | 194.9 | 1.1 | I | P | 222.7 | P | 209.2 | P | -0.6 | D | Trim30 | gb:BM240719 /DB_XREF=gi:17875989 /DB_XREF=K0606D02-3 /CLONE=K0606D02 /FEA=FLmRNA /CNT=122 /TID=Mm.3288.1 /TIER=Stack /STK=26 /UG=Mm.3288 /LL=20128 /UG_GENE=Trim30 /UG_TITLE=tripartite motif protein 30 /FL=gb:BC005447.1 gb:J03776.1 gb:AF220014.1 gb:NM_009099.1 |
| 1418113_at | 559.8 | P | 718.7 | 0.7 | I | P | 466.7 | P | 310.2 | P | -0.8 | D | Cyp2d10 | gb:BC010989.1 /DB_XREF=gi:15012160 /FEA=FLmRNA /CNT=128 /TID=Mm.174372.1 /TIER=FL+Stack /STK=29 /UG=Mm.174372 /LL=13101 /UG_GENE=Cyp2d10 /DEF=Mus musculus, cytochrome P450, 2d10, clone MGC:14081 IMAGE:4193743, mRNA, complete cds. /PROD=cytochrome P450, 2d10 /FL=gb:BC010989.1 gb:M27167.1 gb:NM_010005.1 |
| 1418138_at | 24.6 | A | 137.9 | 2 | I | P | 406.2 | P | 181.9 | P | -1.3 | D | Sultn | gb:AI647561 /DB_XREF=gi:4726239 /DB_XREF=uk41b09.x1 /CLONE=IMAGE:1971545 /FEA=FLmRNA /CNT=100 /TID=Mm.6824.1 /TIER=ConsEnd /STK=0 /UG=Mm.6824 /LL=53315 /UG_GENE=Sultn /UG_TITLE=N-sulfotransferase /FL=gb:U32371.1 gb:NM_016771.1 gb:AF026073.1 gb:BC010752.1 |
| 1418175_at | 158.3 | P | 335.5 | 1 | I | P | 883.9 | P | 430 | P | -1.1 | D | Vdr | gb:BC006716.1 /DB_XREF=gi:13879474 /FEA=FLmRNA /CNT=145 /TID=Mm.44170.1 /TIER=FL+Stack /STK=28 /UG=Mm.44170 /LL=22337 /UG_GENE=Vdr /DEF=Mus musculus, vitamin D receptor, clone MGC:12147 IMAGE:3710866, mRNA, complete cds. /PROD=vitamin D receptor /FL=gb:D31969.1 gb:BC006716.1 gb:NM_009504.1 |
| 1418190_at | 902.6 | P | 1659.5 | 0.9 | I | P | 457.9 | P | 239.7 | P | -1 | D | Pon1 | gb:NM_011134.1 /DB_XREF=gi:7242182 /GEN=Pon1 /FEA=FLmRNA /CNT=79 /TID=Mm.30107.1 /TIER=FL+Stack /STK=28 /UG=Mm.30107 /LL=18979 /DEF=Mus musculus paraoxonase 1 (Pon1), mRNA. /PROD=paraoxonase 1 /FL=gb:U32684.1 gb:NM_011134.1 gb:BC012706.1 gb:U72636.1 |
| 1418282_x_at | 4696.8 | P | 7799.6 | 0.6 | I | P | 4217 | P | 1977.5 | P | -0.8 | D | Spi1-2 | gb:NM_009244.1 /DB_XREF=gi:6678080 /GEN=Spi1-2 /FEA=FLmRNA /CNT=114 /TID=Mm.193418.1 /TIER=FL+Stack /STK=26 /UG=Mm.193418 /LL=20701 /DEF=Mus musculus serine protease inhibitor 1-2 (Spi1-2), mRNA. /PROD=serine protease inhibitor 1-2 /FL=gb:M25529.1 gb:NM_009244.1 |
| 1418627_at | 352.7 | P | 1084.7 | 1.5 | I | P | 848.7 | P | 559.2 | P | -0.9 | D | Gclm | gb:NM_008129.1 /DB_XREF=gi:6680018 /GEN=Gclm /FEA=FLmRNA /CNT=164 /TID=Mm.29340.1 /TIER=FL+Stack /STK=20 /UG=Mm.29340 /LL=14630 /DEF=Mus musculus glutamate-cysteine ligase , modifier subunit (Gclm), mRNA. /PROD=glutamate cysteine ligase(gamma-glutamylcysteine synthetase), regulatory /FL=gb:NM_008129.1 gb:U95053.1 |
| 1418696_at | 406.6 | P | 636.2 | 0.2 | I | P | 1324.9 | P | 328.3 | P | -2 | D | MGC37912 | gb:NM_138951.1 /DB_XREF=gi:20336733 /GEN=MGC37912 /FEA=FLmRNA /CNT=83 /TID=Mm.27244.1 /TIER=FL+Stack /STK=19 /UG=Mm.27244 /LL=192653 /DEF=Mus musculus hypothetical protein MGC37912 (MGC37912), mRNA. /PROD=hypothetical protein MGC37912 /FL=gb:BC021613.1 gb:BC024432.1 gb:BC021608.1 gb:NM_138951.1 gb:BC026825.1 |
| 1418809_at | 74.4 | P | 361.5 | 1.9 | I | P | 300.7 | P | 146.9 | P | -0.6 | D | Pira1 | gb:NM_011087.1 /DB_XREF=gi:6755063 /GEN=Pira1 /FEA=FLmRNA /CNT=25 /TID=Mm.193462.1 /TIER=FL+Stack /STK=18 /UG=Mm.193462 /LL=18722 /DEF=Mus musculus paired-Ig-like receptor A1 (Pira1), mRNA. /PROD=paired-Ig-like receptor A1 /FL=gb:U96682.1 gb:NM_011087.1 |
| 1419093_at | 665.3 | P | 2028.7 | 1.5 | I | P | 511.5 | P | 317.1 | P | -0.8 | D | Tdo2 | gb:BC018390.1 /DB_XREF=gi:17390919 /FEA=FLmRNA /CNT=111 /TID=Mm.21545.1 /TIER=FL+Stack /STK=13 /UG=Mm.21545 /LL=56720 /UG_GENE=Tdo2 /DEF=Mus musculus, tryptophan 2,3-dioxygenase, clone MGC:25811 IMAGE:4159877, mRNA, complete cds. /PROD=tryptophan 2,3-dioxygenase /FL=gb:NM_019911.1 gb:BC018390.1 gb:U24493.1 |
| 1419131_at | 129.1 | P | 241.7 | 1.1 | I | P | 335.5 | P | 116.4 | P | -2 | D | F13b | gb:NM_031164.1 /DB_XREF=gi:13624320 /GEN=F13b /FEA=FLmRNA /CNT=32 /TID=Mm.30105.1 /TIER=FL+Stack /STK=13 /UG=Mm.30105 /LL=14060 /DEF=Mus musculus coagulation factor XIII, beta subunit (F13b), mRNA. /PROD=coagulation factor XIII, beta subunit /FL=gb:D10071.1 gb:NM_031164.1 |
| 1419161_a_at | 52.4 | M | 115 | 1.1 | I | P | 181.2 | P | 104.8 | P | -0.7 | D | Kox-1 | gb:AB041034.1 /DB_XREF=gi:7861540 /GEN=Kox-1 /FEA=FLmRNA /CNT=100 /TID=Mm.31748.1 /TIER=FL+Stack /STK=12 /UG=Mm.31748 /LL=50490 /DEF=Mus musculus Kox-1 mRNA for kidney superoxide-producing NADPH oxidase, complete cds. /PROD=kidney superoxide-producing NADPH oxidase /FL=gb:AF218723.1 gb:AF261944.1 gb:AB041034.1 gb:AF276957.1 gb:NM_015760.1 gb:AB042745.1 |
| 1419196_at | 383.7 | P | 1555.4 | 2.1 | I | P | 757.1 | P | 393.8 | P | -1 | D | Hamp | gb:NM_032541.1 /DB_XREF=gi:14211541 /GEN=Hamp /FEA=FLmRNA /CNT=18 /TID=Mm.23995.1 /TIER=FL+Stack /STK=12 /UG=Mm.23995 /LL=84506 /DEF=Mus musculus hepcidin antimicrobial peptide (Hamp), mRNA. /PROD=hepcidin antimicrobial peptide /FL=gb:NM_032541.1 gb:AF297664.1 gb:BC021587.1 |
| 1419349_a_at | 211 | P | 737.9 | 2 | I | P | 703.9 | P | 257.2 | P | -1.5 | D | Cyp2d9 | gb:BC010593.1 /DB_XREF=gi:14714876 /FEA=FLmRNA /CNT=127 /TID=Mm.104363.1 /TIER=FL+Stack /STK=10 /UG=Mm.104363 /LL=13105 /UG_GENE=Cyp2d9 /DEF=Mus musculus, cytochrome P450, 2d9, clone MGC:11540 IMAGE:3964178, mRNA, complete cds. /PROD=cytochrome P450, 2d9 /FL=gb:BC010593.1 gb:M23998.1 gb:M27168.1 gb:NM_010006.1 |
| 1419622_at | 159.9 | P | 248 | 0.5 | I | P | 295.4 | P | 26.8 | A | -2.9 | D | Ugt2b5 | gb:NM_009467.1 /DB_XREF=gi:6678500 /GEN=Ugt2b5 /FEA=FLmRNA /CNT=11 /TID=Mm.29157.1 /TIER=FL+Stack /STK=8 /UG=Mm.29157 /LL=22238 /DEF=Mus musculus UDP-glucuronosyltransferase 2 family, member 5 (Ugt2b5), mRNA. /PROD=UDP-glucuronosyltransferase 2 family, member 5 /FL=gb:NM_009467.1 |
| 1419914_s_at | 826 | P | 1264.9 | 0.4 | I | P | 1579 | P | 1067.8 | P | -0.7 | D | AW538011 | gb:AW538011 /DB_XREF=gi:7180428 /DB_XREF=C0102B03-3 /CLONE=C0102B03 /FEA=EST /CNT=1 /TID=Mm.199964.1 /TIER=ConsEnd /STK=1 /UG=Mm.199964 /LL=103464 /UG_GENE=AW538011 /UG_TITLE=expressed sequence AW538011 |
| 1420553_x_at | 2913.9 | P | 4719.8 | 0.7 | I | P | 1866.3 | P | 1331.9 | P | -0.6 | D | Spi1-1 | gb:NM_009243.1 /DB_XREF=gi:6678078 /GEN=Spi1-1 /FEA=FLmRNA /CNT=10 /TID=Mm.218230.1 /TIER=FL /STK=4 /UG=Mm.218230 /LL=20700 /DEF=Mus musculus serine protease inhibitor 1-1 (Spi1-1), mRNA. /PROD=serine protease inhibitor 1-1 /FL=gb:M75721.1 gb:NM_009243.1 |
| 1420760_s_at | 795.2 | P | 1935.1 | 1.1 | I | P | 5385 | P | 2732.9 | P | -0.7 | D | Ndrl | gb:NM_008681.1 /DB_XREF=gi:6679029 /GEN=Ndrl /FEA=FLmRNA /CNT=5 /TID=Mm.30837.1 /TIER=FL /STK=3 /UG=Mm.30837 /LL=17990 /DEF=Mus musculus N-myc downstream regulated-like (Ndrl), mRNA. /PROD=N-myc downstream regulated-like /FL=gb:NM_008681.1 gb:U52073.1 |
| 1420836_at | 158.6 | P | 721.3 | 2 | I | P | 1005.3 | P | 571 | P | -0.7 | D | 4933433D23Rik | gb:BB032012 /DB_XREF=gi:15403590 /DB_XREF=BB032012 /CLONE=5830468I05 /FEA=FLmRNA /CNT=155 /TID=Mm.41116.1 /TIER=Stack /STK=112 /UG=Mm.41116 /LL=67554 /UG_GENE=4933433D23Rik /UG_TITLE=RIKEN cDNA 4933433D23 gene /FL=gb:NM_026232.1 |
| 1420941_at | 43.5 | A | 776.1 | 4.8 | I | P | 272.2 | P | 134.9 | P | -1.8 | D | Rgs5 | gb:AK004165.1 /DB_XREF=gi:12835246 /GEN=Rgs5 /FEA=FLmRNA /CNT=77 /TID=Mm.20954.1 /TIER=Stack /STK=36 /UG=Mm.20954 /LL=19737 /UG_TITLE=regulator of G-protein signaling 5 /DEF=Mus musculus 18 days embryo whole body cDNA, RIKEN full-length enriched library, clone:1110038N04:regulator of G-protein signaling 5, full insert sequence. /FL=gb:U67188.1 gb:NM_009063.1 |
| 1421009_at | 304.4 | P | 600.9 | 1.1 | I | P | 692.1 | P | 174 | P | -1.9 | D | Vig1 | gb:BB741897 /DB_XREF=gi:16144902 /DB_XREF=BB741897 /CLONE=F520004L04 /FEA=FLmRNA /CNT=44 /TID=Mm.24045.1 /TIER=Stack /STK=10 /UG=Mm.24045 /LL=58185 /UG_GENE=Vig1-pending /UG_TITLE=viral hemorrhagic septicemia virus(VHSV) induced gene 1 /FL=gb:NM_021384.1 gb:AF442152.1 gb:AF236064.2 |
| 1421217_a_at | 341.1 | P | 704.5 | 0.7 | I | P | 333.1 | P | 153.4 | P | -1 | D | Lgals9 | gb:NM_010708.1 /DB_XREF=gi:6754535 /GEN=Lgals9 /FEA=FLmRNA /CNT=14 /TID=Mm.18087.1 /TIER=FL /STK=2 /UG=Mm.18087 /LL=16859 /DEF=Mus musculus lectin, galactose binding, soluble 9 (Lgals9), mRNA. /PROD=lectin, galactose binding, soluble 9 /FL=gb:U55061.1 gb:NM_010708.1 |
| 1422444_at | 104.3 | A | 443.2 | 2.2 | I | P | 595 | P | 343.9 | P | -0.9 | D | Itga6 | gb:NM_008397.1 /DB_XREF=gi:7110658 /GEN=Itga6 /FEA=FLmRNA /CNT=336 /TID=Mm.25232.1 /TIER=FL+Stack /STK=112 /UG=Mm.25232 /LL=16403 /DEF=Mus musculus integrin alpha 6 (Itga6), mRNA. /PROD=integrin alpha 6 /FL=gb:NM_008397.1 |
| 1423439_at | 712 | P | 1637.8 | 1 | I | P | 2364.1 | P | 669.3 | P | -2 | D | Pck1 | gb:AW106963 /DB_XREF=gi:6077763 /DB_XREF=um34h07.x1 /CLONE=IMAGE:2236477 /FEA=FLmRNA /CNT=259 /TID=Mm.42246.1 /TIER=Stack /STK=25 /UG=Mm.42246 /LL=18534 /UG_GENE=Pck1 /UG_TITLE=phosphoenolpyruvate carboxykinase 1, cytosolic /FL=gb:NM_011044.1 |
| 1423523_at | 161.1 | P | 687 | 2.1 | I | P | 955.8 | P | 292.6 | P | -1.7 | D | Lorsdh | gb:BF687395 /DB_XREF=gi:11972803 /DB_XREF=602102567F1 /CLONE=IMAGE:4220560 /FEA=FLmRNA /CNT=91 /TID=Mm.18651.1 /TIER=Stack /STK=13 /UG=Mm.18651 /LL=30956 /UG_GENE=Lorsdh /UG_TITLE=lysine oxoglutarate reductase, saccharopine dehydrogenase /FL=gb:NM_013930.1 gb:BC005420.1 |
| 1423569_at | 100.4 | A | 177.9 | 1 | I | P | 349.7 | P | 110.2 | P | -1.6 | D | Gatm | gb:AW108522 /DB_XREF=gi:6079322 /DB_XREF=um31e05.x1 /CLONE=IMAGE:2236160 /FEA=FLmRNA /CNT=271 /TID=Mm.29975.1 /TIER=Stack /STK=10 /UG=Mm.29975 /LL=67092 /UG_GENE=Gatm /UG_TITLE=glycine amidinotransferase (L-arginine:glycine amidinotransferase) /FL=gb:BC003879.1 gb:NM_025961.1 |
| 1424400_a_at | 381 | P | 622.1 | 0.5 | I | P | 825.9 | P | 327.2 | P | -1.4 | D | 1810048F20Rik | gb:BC025939.1 /DB_XREF=gi:19684150 /FEA=FLmRNA /CNT=116 /TID=Mm.30035.1 /TIER=FL+Stack /STK=34 /UG=Mm.30035 /LL=70374 /UG_GENE=1810048F20Rik /DEF=Mus musculus, Similar to 10-formyltetrahydrofolate dehydrogenase, clone MGC:37834 IMAGE:5099360, mRNA, complete cds. /PROD=Similar to 10-formyltetrahydrofolatedehydrogenase /FL=gb:BC025939.1 |
| 1424783_a_at | 998 | P | 1510.4 | 0.6 | I | P | 2267.4 | P | 955.5 | P | -0.9 | D | Ugt1a1 | gb:BC019434.1 /DB_XREF=gi:18043822 /FEA=FLmRNA /CNT=66 /TID=Mm.42472.1 /TIER=FL+Stack /STK=16 /UG=Mm.42472 /LL=22236 /UG_GENE=Ugt1a1 /DEF=Mus musculus, UDP-glucuronosyltransferase 1 family, member 1, clone MGC:30240 IMAGE:5126634, mRNA, complete cds. /PROD=UDP-glucuronosyltransferase 1 family, member 1 /FL=gb:BC019434.1 gb:L27122.1 |
| 1424893_at | 122.4 | M | 292.2 | 1.3 | I | P | 293.3 | P | 189.3 | P | -0.8 | D | Nudel | gb:BC021434.1 /DB_XREF=gi:18204045 /FEA=FLmRNA /CNT=20 /TID=Mm.31979.2 /TIER=FL+Stack /STK=12 /UG=Mm.31979 /LL=83431 /UG_GENE=Nudel-pending /DEF=Mus musculus, Similar to nuclear distribution gene E-like, clone MGC:29336 IMAGE:5029832, mRNA, complete cds. /PROD=Similar to nuclear distribution gene E-like /FL=gb:BC021434.1 |
| 1424898_at | 255.1 | P | 659 | 1.3 | I | P | 143.8 | P | 102.9 | P | -1 | D | Slc10a1 | gb:BC021154.1 /DB_XREF=gi:18088160 /FEA=FLmRNA /CNT=28 /TID=Mm.104295.2 /TIER=FL+Stack /STK=12 /UG=Mm.104295 /LL=20493 /UG_GENE=Slc10a1 /DEF=Mus musculus, Similar to Solute carrier family 10 (sodiumbile acid cotransporter family), member 1, clone MGC:13910 IMAGE:4162098, mRNA, complete cds. /PROD=Similar to Solute carrier family 10 (sodiumbileacid cotransporter family), member 1 /FL=gb:AB003303.1 gb:U95131.1 gb:BC021154.1 |
| 1425140_at | 225.7 | P | 378 | 0.8 | I | P | 636.2 | P | 253.1 | P | -1.4 | D | BC004045 | gb:BC004045.1 /DB_XREF=gi:13278494 /FEA=FLmRNA /CNT=119 /TID=Mm.89572.1 /TIER=FL /STK=5 /UG=Mm.89572 /DEF=Mus musculus, clone MGC:7674 IMAGE:3496398, mRNA, complete cds. /PROD=Unknown (protein for MGC:7674) /FL=gb:BC004045.1 |
| 1425451_s_at | 234.8 | P | 776.4 | 1.5 | I | P | 290.3 | P | 120.2 | P | -1.3 | D | Ym2 | gb:AY065557.1 /DB_XREF=gi:18086513 /GEN=Ym2 /FEA=FLmRNA /CNT=2 /TID=Mm.215253.1 /TIER=FL /STK=2 /UG=Mm.215253 /DEF=Mus musculus putative secretory protein precursor (Ym2) mRNA, complete cds. /PROD=putative secretory protein precursor /FL=gb:AY065557.1 gb:AY049765.1 |
| 1426219_at | 2399.4 | P | 2818.8 | 0.2 | I | P | 2860.9 | P | 1533.6 | P | -0.9 | D | Scp2 | gb:M62361.1 /DB_XREF=gi:200941 /FEA=FLmRNA /CNT=466 /TID=Mm.1779.1 /TIER=FL+Stack /STK=49 /UG=Mm.1779 /LL=20280 /UG_GENE=Scp2 /DEF=Mus musculus sterol carrier protein-2 (SCP-2) gene, complete cds. /PROD=sterol carrier protein-2 /FL=gb:M91458.1 gb:BC018384.1 gb:M62361.1 |
| 1426252_a_at | 156.9 | P | 282.5 | 1.1 | I | P | 4107.5 | P | 1986.2 | P | -0.9 | D | Umod | gb:BC012973.1 /DB_XREF=gi:15278016 /FEA=FLmRNA /CNT=290 /TID=Mm.10826.1 /TIER=FL+Stack /STK=23 /UG=Mm.10826 /LL=22242 /UG_GENE=Umod /DEF=Mus musculus, Similar to uromodulin, clone MGC:14034 IMAGE:4210940, mRNA, complete cds. /PROD=Similar to uromodulin /FL=gb:L33406.1 gb:BC012973.1 |
| 1426260_a_at | 1500 | P | 2786.6 | 0.9 | I | P | 3565.2 | P | 1718.3 | P | -1.1 | D | UGP1a1 | gb:D87867.1 /DB_XREF=gi:1660991 /GEN=UGP1a1 /FEA=FLmRNA /CNT=20 /TID=Mm.42472.3 /TIER=FL+Stack /STK=19 /UG=Mm.42472 /LL=22236 /DEF=Mus musculus mRNA for phenol UDP-glucuronosyltransferase, complete cds. /PROD=phenol UDP-glucuronosyltransferase /FL=gb:D87867.1 |
| 1426261_s_at | 355.8 | P | 929.5 | 1.4 | I | P | 878.1 | P | 504.4 | P | -0.9 | D | UGP1a1 | gb:D87867.1 /DB_XREF=gi:1660991 /GEN=UGP1a1 /FEA=FLmRNA /CNT=20 /TID=Mm.42472.3 /TIER=FL+Stack /STK=19 /UG=Mm.42472 /LL=22236 /DEF=Mus musculus mRNA for phenol UDP-glucuronosyltransferase, complete cds. /PROD=phenol UDP-glucuronosyltransferase /FL=gb:D87867.1 |
| 1426438_at | 50.2 | P | 373.4 | 2.8 | I | P | 2334 | P | 927.1 | P | -1.3 | D | Dby | gb:BB667072 /DB_XREF=gi:16398521 /DB_XREF=BB667072 /CLONE=2610028H19 /FEA=FLmRNA /CNT=123 /TID=Mm.20950.1 /TIER=Stack /STK=19 /UG=Mm.20950 /LL=26900 /UG_GENE=Dby /UG_TITLE=DEAD (aspartate-glutamate-alanine-aspartate) box polypeptide, Y chromosome /FL=gb:BC021453.1 |
| 1427000_at | 69.9 | P | 173.2 | 1.5 | I | P | 402.6 | P | 86.5 | P | -2.1 | D | BF580781 | gb:BF580781 /DB_XREF=gi:11654493 /DB_XREF=602093939F1 /CLONE=IMAGE:4208433 /FEA=mRNA /CNT=63 /TID=Mm.202383.1 /TIER=Stack /STK=27 /UG=Mm.202383 /UG_TITLE=Mus musculus, clone IMAGE:4990763, mRNA |
| 1427126_at | 157.7 | P | 327 | 0.5 | I | P | 578.1 | P | 201.9 | P | -1.3 | D | Hsp70-1 | gb:M12573.1 /DB_XREF=gi:194018 /FEA=mRNA /CNT=23 /TID=Mm.6388.1 /TIER=Stack /STK=15 /UG=Mm.6388 /LL=15511 /UG_GENE=Hsp70-1 /UG_TITLE=heat shock protein, 70 kDa 1 /DEF=Mouse heat shock protein (hsp68) mRNA, clone MHS214, partial cds. |
| 1427337_at | 143.6 | P | 275.8 | 0.7 | I | P | 387.2 | P | 110.5 | P | -1.9 | D | aldehyde dehydrogenase 8 family, member A1 | gb:BC013511.1 /DB_XREF=gi:15488741 /FEA=mRNA /CNT=30 /TID=Mm.90181.1 /TIER=ConsEnd /STK=3 /UG=Mm.90181 /DEF=Mus musculus, Similar to aldehyde dehydrogenase 8 family, member A1, clone IMAGE:4234742, mRNA, partial cds. /PROD=Similar to aldehyde dehydrogenase 8 family,member A1 |
| 1427345_a_at | 664.6 | P | 868.7 | 0.3 | I | P | 470.5 | P | 188.4 | P | -0.7 | D | Sult1a1 | gb:AK002700.1 /DB_XREF=gi:12832875 /GEN=Sult1a1 /FEA=mRNA /CNT=24 /TID=Mm.17339.2 /TIER=ConsEnd /STK=6 /UG=Mm.17339 /LL=20887 /UG_TITLE=sulfotransferase family 1A, phenol-preferring, member 1 /DEF=Mus musculus adult male kidney cDNA, RIKEN full-length enriched library, clone:0610030E04:sulfotransferase family 1A, phenol-preferring, member 1, full insert sequence. |
| 1427486_at | 466.6 | P | 634.8 | 0.2 | I | P | 800.2 | P | 365.5 | P | -1 | D | Ptprb | gb:AF157628.1 /DB_XREF=gi:8886020 /FEA=mRNA /CNT=12 /TID=Mm.37213.1 /TIER=ConsEnd /STK=0 /UG=Mm.37213 /LL=19263 /UG_GENE=Ptprb /DEF=Mus musculus receptor-type protein tyrosine phosphatase mRNA, partial cds. /PROD=receptor-type protein tyrosine phosphatase |
| 1428853_at | 927.4 | P | 1564.7 | 0.7 | I | P | 2220.6 | P | 1222.1 | P | -0.6 | D | A230106A15Rik | gb:BG071079 /DB_XREF=gi:12553648 /DB_XREF=H3094E02-3 /CLONE=H3094E02 /FEA=mRNA /CNT=57 /TID=Mm.200220.1 /TIER=Stack /STK=19 /UG=Mm.200220 /LL=77214 /UG_GENE=A230106A15Rik /UG_TITLE=RIKEN cDNA A230106A15 gene |
| 1429775_a_at | 335.2 | P | 450 | 0.6 | I | P | 418.7 | P | 226.5 | P | -0.8 | D | Tm7sf1 | gb:AK009736.1 /DB_XREF=gi:12844712 /FEA=mRNA /CNT=16 /TID=Mm.1585.2 /TIER=Stack /STK=8 /UG=Mm.1585 /LL=83924 /UG_GENE=Tm7sf1 /UG_TITLE=transmembrane 7 superfamily member 1 /DEF=Mus musculus adult male tongue cDNA, RIKEN full-length enriched library, clone:2310041G17:similar to PUTATIVE SEVEN PASS TRANSMEMBRANE PROTEIN, full insert sequence. |
| 1431328_at | 327.4 | P | 506.2 | 0.9 | I | P | 617.6 | P | 350.5 | P | -0.9 | D | Ppp1cb | gb:AK017392.1 /DB_XREF=gi:12856610 /FEA=mRNA /CNT=5 /TID=Mm.4572.2 /TIER=ConsEnd /STK=0 /UG=Mm.4572 /LL=19046 /UG_GENE=Ppp1cb /UG_TITLE=protein phosphatase 1, catalytic subunit, beta isoform /DEF=Mus musculus 6 days neonate head cDNA, RIKEN full-length enriched library, clone:5430434K07:protein phosphatase 1, catalytic subunit, beta isoform, full insert sequence. |
| 1433942_at | 254.4 | P | 380.4 | 0.7 | I | P | 479.4 | P | 338.9 | P | -0.6 | D | BE133806 | gb:BE133806 /DB_XREF=gi:8596238 /DB_XREF=ug11d04.x1 /CLONE=IMAGE:1531303 /FEA=EST /CNT=64 /TID=Mm.215129.1 /TIER=Stack /STK=50 /UG=Mm.215129 /UG_TITLE=ESTs |
| 1435162_at | 162 | P | 301.9 | 0.9 | I | P | 440.4 | P | 259.9 | P | -0.9 | D | Prkg2 | gb:BB823350 /DB_XREF=gi:17001593 /DB_XREF=BB823350 /CLONE=G830027G08 /FEA=EST /CNT=33 /TID=Mm.44410.2 /TIER=Stack /STK=19 /UG=Mm.44410 /LL=19092 /UG_GENE=Prkg2 /UG_TITLE=protein kinase, cGMP-dependent, type II |
| 1435602_at | 176.7 | P | 998.1 | 2.1 | I | P | 1461.7 | P | 635.7 | P | -1.3 | D | Sps2 | gb:BE200310 /DB_XREF=gi:8712407 /DB_XREF=ug74e02.x1 /CLONE=IMAGE:1548122 /FEA=EST /CNT=17 /TID=Mm.20294.2 /TIER=Stack /STK=15 /UG=Mm.20294 /LL=20768 /UG_GENE=Sps2 /UG_TITLE=selenophosphate synthetase 2 |
| 1436746_at | 191.2 | P | 556.2 | 1.2 | I | P | 811.7 | P | 544.2 | P | -0.9 | D | kinase, lysine deficient 1 | gb:BI692255 /DB_XREF=gi:15654884 /DB_XREF=603342721F1 /CLONE=IMAGE:5370700 /FEA=EST /CNT=96 /TID=Mm.27341.1 /TIER=Stack /STK=69 /UG=Mm.27341 /UG_TITLE=ESTs, Moderately similar to protein kinase, lysine deficient 1 (Rattus norvegicus) (R.norvegicus) |
| 1437110_at | 71 | A | 494.5 | 2.9 | I | P | 351.3 | P | 166.6 | P | -0.8 | D | BM232998 | gb:BM232998 /DB_XREF=gi:17868268 /DB_XREF=K0331A04-3 /CLONE=K0331A04 /FEA=EST /CNT=48 /TID=Mm.103435.2 /TIER=Stack /STK=25 /UG=Mm.103435 /UG_TITLE=Mus musculus, clone IMAGE:4952280, mRNA |
| 1437667_a_at | 37.7 | P | 134.9 | 1.6 | I | P | 232.3 | P | 102.3 | P | -1.2 | D | Bach2 | gb:AW553304 /DB_XREF=gi:7198727 /DB_XREF=L0225B12-3 /CLONE=L0225B12 /FEA=EST /CNT=17 /TID=Mm.21908.5 /TIER=Stack /STK=15 /UG=Mm.21908 /LL=12014 /UG_GENE=Bach2 /UG_TITLE=BTB and CNC homology 2 |
| 1438009_at | 3092.7 | P | 5961.8 | 1.2 | I | P | 3504.9 | P | 2089.4 | P | -0.8 | D | H2afx | gb:W91024 /DB_XREF=gi:1541291 /DB_XREF=mf83h06.r1 /CLONE=IMAGE:420923 /FEA=EST /CNT=79 /TID=Mm.14767.2 /TIER=Stack /STK=12 /UG=Mm.14767 /LL=15270 /UG_GENE=H2afx /UG_TITLE=H2A histone family, member X |
| 1438069_a_at | 41.8 | A | 104.2 | 1.4 | I | P | 183.4 | P | 42.6 | A | -1.7 | D | BE446879 | gb:BE446879 /DB_XREF=gi:9446487 /DB_XREF=us88g05.x1 /CLONE=IMAGE:3325496 /FEA=EST /CNT=19 /TID=Mm.46706.2 /TIER=Stack /STK=12 /UG=Mm.46706 /UG_TITLE=Mus musculus, clone IMAGE:3491703, mRNA, partial cds |
| 1448452_at | 101.9 | A | 239.3 | 1.1 | I | P | 218.7 | A | 115.9 | A | -1.1 | D | Icsbp | gb:BG069095 /DB_XREF=gi:12551664 /DB_XREF=H3072F09-3 /CLONE=H3072F09 /FEA=FLmRNA /CNT=157 /TID=Mm.3182.1 /TIER=Stack /STK=20 /UG=Mm.3182 /LL=15900 /UG_GENE=Icsbp /UG_TITLE=interferon concensus sequence binding protein /FL=gb:BC005450.1 gb:M32489.1 gb:NM_008320.1 |
| 1448470_at | 1317.4 | P | 2344.4 | 0.9 | I | P | 5481.2 | P | 894.1 | P | -2.7 | D | Fbp1 | gb:NM_019395.1 /DB_XREF=gi:9506588 /GEN=Fbp1 /FEA=FLmRNA /CNT=228 /TID=Mm.30154.1 /TIER=FL+Stack /STK=68 /UG=Mm.30154 /LL=14121 /DEF=Mus musculus fructose bisphosphatase 1 (Fbp1), mRNA. /PROD=fructose bisphosphatase 1 /FL=gb:NM_019395.1 gb:BC011480.1 |
| 1448525_a_at | 353.7 | P | 890.6 | 1.4 | I | P | 577.3 | P | 217.7 | P | -1 | D | Bnip3l | gb:AK018668.1 /DB_XREF=gi:12858490 /GEN=Bnip3l /FEA=FLmRNA /CNT=212 /TID=Mm.29820.1 /TIER=Stack /STK=9 /UG=Mm.29820 /LL=12177 /UG_TITLE=BCL2adenovirus E1B 19 kDa-interacting protein 3-like /DEF=Mus musculus adult male cecum cDNA, RIKEN full-length enriched library, clone:9130410L09:BCL2adenovirus E1B 19 kDa-interacting protein 3-like, full insert sequence. /FL=gb:NM_009761.1 gb:AF067395.1 |
| 1448939_at | 225.8 | P | 445.2 | 0.7 | I | P | 277 | P | 149.4 | P | -1.1 | D | Usp25 | gb:NM_013918.1 /DB_XREF=gi:7305616 /GEN=Usp25 /FEA=FLmRNA /CNT=132 /TID=Mm.40986.1 /TIER=FL+Stack /STK=30 /UG=Mm.40986 /LL=30940 /DEF=Mus musculus ubiquitin specific protease 25 (Usp25), mRNA. /PROD=ubiquitin specific protease 25 /FL=gb:NM_013918.1 gb:AF170563.1 |
| 1448973_at | 395.3 | P | 499.1 | 0.9 | I | P | 1859.6 | P | 763.2 | P | -1.7 | D | Sultn | gb:NM_016771.1 /DB_XREF=gi:7949145 /GEN=Sultn /FEA=FLmRNA /CNT=100 /TID=Mm.6824.1 /TIER=FL+Stack /STK=29 /UG=Mm.6824 /LL=53315 /DEF=Mus musculus N-sulfotransferase (Sultn), mRNA. /PROD=N-sulfotransferase /FL=gb:U32371.1 gb:NM_016771.1 gb:AF026073.1 gb:BC010752.1 |
| 1449269_at | 248.4 | P | 549.3 | 0.7 | I | P | 460.1 | P | 225.9 | P | -1.1 | D | F5 | gb:NM_007976.1 /DB_XREF=gi:6679730 /GEN=F5 /FEA=FLmRNA /CNT=57 /TID=Mm.12900.1 /TIER=FL+Stack /STK=16 /UG=Mm.12900 /LL=14067 /DEF=Mus musculus coagulation factor V (F5), mRNA. /PROD=coagulation factor V /FL=gb:NM_007976.1 gb:U52925.1 |
| 1449374_at | 115.8 | P | 225 | 0.5 | I | P | 696.5 | P | 153.1 | P | -1.8 | D | Pso | gb:BC013525.1 /DB_XREF=gi:15488781 /FEA=FLmRNA /CNT=74 /TID=Mm.8543.1 /TIER=FL+Stack /STK=12 /UG=Mm.8543 /LL=19193 /UG_GENE=Pso /DEF=Mus musculus, peroxisomal sarcosine oxidase, clone MGC:19202 IMAGE:4237443, mRNA, complete cds. /PROD=peroxisomal sarcosine oxidase /FL=gb:BC013525.1 gb:NM_008952.1 gb:U94700.1 |
| 1450185_a_at | 38 | A | 77.1 | 0.7 | I | P | 292.4 | P | 65 | P | -1.8 | D | Kcnj15 | gb:NM_019664.1 /DB_XREF=gi:9790078 /GEN=Kcnj15 /FEA=FLmRNA /CNT=11 /TID=Mm.44238.1 /TIER=FL /STK=2 /UG=Mm.44238 /LL=16516 /DEF=Mus musculus potassium inwardly-rectifying channel, subfamily J, member 15 (Kcnj15), mRNA. /PROD=potassium inwardly-rectifying channel, subfamilyJ, member 15 /FL=gb:AF085696.1 gb:NM_019664.1 |
| 1450624_at | 1175.9 | P | 1494.6 | 0.3 | I | P | 683 | P | 267.5 | P | -1.3 | D | Bhmt | gb:NM_016668.1 /DB_XREF=gi:7709989 /GEN=Bhmt /FEA=FLmRNA /CNT=286 /TID=Mm.21983.1 /TIER=FL+Stack /STK=137 /UG=Mm.21983 /LL=12116 /DEF=Mus musculus betaine-homocysteine methyltransferase (Bhmt), mRNA. /PROD=betaine-homocysteine methyltransferase /FL=gb:AF033381.1 gb:NM_016668.1 |
| 1450634_at | 273.6 | P | 778.2 | 1.5 | I | P | 337.4 | P | 241.3 | P | -0.7 | D | Atp6v1a1 | gb:NM_007508.1 /DB_XREF=gi:6680751 /GEN=Atp6v1a1 /FEA=FLmRNA /CNT=338 /TID=Mm.29771.1 /TIER=FL+Stack /STK=62 /UG=Mm.29771 /LL=11964 /DEF=Mus musculus ATPase, H+ transporting, lysosomal 70kD, V1 subunit A, isoform 1 (Atp6v1a1), mRNA. /PROD=ATPase, H+ transporting, lysosomal 70kD, V1subunit A, isoform 1 /FL=gb:NM_007508.1 |
| 1450715_at | 197.3 | P | 589.1 | 0.9 | I | P | 364.3 | P | 120.6 | P | -1.3 | D | Cyp1a2 | gb:NM_009993.1 /DB_XREF=gi:6753565 /GEN=Cyp1a2 /FEA=FLmRNA /CNT=138 /TID=Mm.15537.1 /TIER=FL+Stack /STK=17 /UG=Mm.15537 /LL=13077 /DEF=Mus musculus cytochrome P450, 1a2, aromatic compound inducible (Cyp1a2), mRNA. /PROD=cytochrome P450, 1a2, aromatic compoundinducible /FL=gb:NM_009993.1 gb:BC018298.1 |
| 1450966_at | 381.3 | P | 1000.2 | 1.5 | I | P | 696.9 | P | 476 | P | -0.7 | D | Crot | gb:BB283187 /DB_XREF=gi:8983636 /DB_XREF=BB283187 /CLONE=A930101D22 /FEA=FLmRNA /CNT=134 /TID=Mm.28197.1 /TIER=Stack /STK=9 /UG=Mm.28197 /LL=74114 /UG_GENE=Crot /UG_TITLE=carnitine O-octanoyltransferase /FL=gb:BC012308.1 gb:NM_023733.1 gb:BC006593.1 |
| 1451071_a_at | 2708.4 | P | 3437.4 | 0.4 | I | P | 8187.4 | P | 5595.7 | P | -0.5 | D | BC021496 | gb:BC025618.1 /DB_XREF=gi:19343735 /FEA=FLmRNA /CNT=513 /TID=Mm.205791.1 /TIER=FL+Stack /STK=208 /UG=Mm.205791 /DEF=Mus musculus, clone MGC:38134 IMAGE:5320976, mRNA, complete cds. /PROD=Unknown (protein for MGC:38134) /FL=gb:BC025811.1 gb:BC025618.1 gb:BC025627.1 gb:BC021496.1 |
| 1451131_at | 205.1 | P | 594.4 | 1.5 | I | P | 616.5 | P | 459.3 | P | -0.5 | D | Aip-1 | gb:AF133669.1 /DB_XREF=gi:4927191 /GEN=Aip-1 /FEA=FLmRNA /CNT=334 /TID=Mm.29924.1 /TIER=FL+Stack /STK=15 /UG=Mm.29924 /LL=54208 /DEF=Mus musculus ARL-6 interacting protein-1 (Aip-1) mRNA, complete cds. /PROD=ARL-6 interacting protein-1 /FL=gb:AF223953.1 gb:AF133669.1 gb:BC010196.1 |
| 1451194_at | 1305.4 | P | 2865.4 | 1.5 | I | P | 7392.8 | P | 1726.6 | P | -2.1 | D | BC016435 | gb:BC024112.1 /DB_XREF=gi:18848232 /FEA=FLmRNA /CNT=473 /TID=Mm.218862.1 /TIER=FL+Stack /STK=69 /UG=Mm.218862 /DEF=Mus musculus, clone MGC:36398 IMAGE:5102118, mRNA, complete cds. /PROD=Unknown (protein for MGC:36398) /FL=gb:BC026577.1 gb:BC024112.1 gb:BC022113.1 gb:BC016435.1 |
| 1451506_at | 574.1 | P | 1611.8 | 1.3 | I | P | 1588.3 | P | 959.8 | P | -0.9 | D | Mef2c | gb:BC026841.1 /DB_XREF=gi:20070855 /FEA=FLmRNA /CNT=127 /TID=Mm.24001.2 /TIER=FL+Stack /STK=13 /UG=Mm.24001 /LL=17260 /UG_GENE=Mef2c /DEF=Mus musculus, Similar to MADS box transcription enhancer factor 2, polypeptide C (myocyte enhancer factor 2C), clone MGC:25468 IMAGE:4481227, mRNA, complete cds. /PROD=Similar to MADS box transcription enhancerfactor 2, polypeptide C (myocyte enhancer factor 2C) /FL=gb:BC026841.1 |
| 1451513_x_at | 5139.4 | P | 7211.7 | 0.5 | I | P | 3658 | P | 2080.4 | P | -0.8 | D | Spi1-2 | gb:BC012874.1 /DB_XREF=gi:15277552 /FEA=FLmRNA /CNT=20 /TID=Mm.193418.2 /TIER=FL+Stack /STK=13 /UG=Mm.193418 /LL=20701 /UG_GENE=Spi1-2 /DEF=Mus musculus, Similar to serine protease inhibitor 1-2, clone MGC:18593 IMAGE:4194027, mRNA, complete cds. /PROD=Similar to serine protease inhibitor 1-2 /FL=gb:BC012874.1 |
| 1451547_at | 78.8 | A | 141.3 | 0.8 | I | P | 262.4 | P | 117.9 | P | -1.1 | D | 0610009A07Rik | gb:BC023358.1 /DB_XREF=gi:19483966 /FEA=FLmRNA /CNT=22 /TID=Mm.24153.1 /TIER=FL+Stack /STK=10 /UG=Mm.24153 /LL=70337 /UG_GENE=0610009A07Rik /DEF=Mus musculus, RIKEN cDNA 0610009A07 gene, clone MGC:32506 IMAGE:5064638, mRNA, complete cds. /PROD=RIKEN cDNA 0610009A07 gene /FL=gb:BC023358.1 |
| 1451559_a_at | 422.8 | P | 783.9 | 0.6 | I | P | 641.4 | P | 351.2 | P | -0.9 | D | mouNRDR | gb:AB045132.1 /DB_XREF=gi:11559413 /GEN=mouNRDR /FEA=FLmRNA /CNT=40 /TID=Mm.27427.2 /TIER=FL+Stack /STK=9 /UG=Mm.27427 /LL=28200 /DEF=Mus musculus mouNRDR mRNA for NADPH-dependent retinol dehydrogenasereductase, complete cds. /PROD=NADPH-dependent retinol dehydrogenasereductase /FL=gb:BC003484.1 gb:AB045132.1 |
| 1451577_at | 72.6 | A | 258 | 1.6 | I | A | 574.5 | A | 250.2 | A | -1.2 | D | Zfp288 | gb:AW491109 /DB_XREF=gi:7061390 /DB_XREF=UI-M-BH3-atw-g-06-0-UI.s1 /CLONE=UI-M-BH3-atw-g-06-0-UI /FEA=FLmRNA /CNT=26 /TID=Mm.38250.2 /TIER=ConsEnd /STK=4 /UG=Mm.38250 /LL=56490 /UG_GENE=Zfp288 /UG_TITLE=zinc finger protein 288 /FL=gb:AF194030.1 |
| 1451827_a_at | 93.6 | P | 237.2 | 1.4 | I | P | 276 | P | 146.5 | P | -0.7 | D | Nox4 | gb:BC021378.1 /DB_XREF=gi:18204713 /FEA=FLmRNA /CNT=5 /TID=Mm.31748.2 /TIER=FL /STK=1 /UG=Mm.31748 /LL=50490 /UG_GENE=Nox4 /DEF=Mus musculus, clone MGC:29375 IMAGE:5044835, mRNA, complete cds. /PROD=Unknown (protein for MGC:29375) /FL=gb:BC021378.1 |
| 1452318_a_at | 400.1 | P | 403.2 | 0.2 | I | P | 1486.2 | P | 631.5 | P | -1.2 | D | Hsp70-1 | gb:M12573.1 /DB_XREF=gi:194018 /FEA=mRNA /CNT=23 /TID=Mm.6388.1 /TIER=Stack /STK=15 /UG=Mm.6388 /LL=15511 /UG_GENE=Hsp70-1 /UG_TITLE=heat shock protein, 70 kDa 1 /DEF=Mouse heat shock protein (hsp68) mRNA, clone MHS214, partial cds. |
| 1452986_at | 427.4 | P | 607 | 0.6 | I | P | 1227.8 | P | 373 | P | -2.2 | D | AK002584 | gb:AK002584.1 /DB_XREF=gi:12832674 /FEA=mRNA /CNT=57 /TID=Mm.157442.1 /TIER=Stack /STK=15 /UG=Mm.157442 /UG_TITLE=Mus musculus adult male kidney cDNA, RIKEN full-length enriched library, clone:0610012C11:homogentisate 1, 2-dioxygenase, full insert sequence /DEF=Mus musculus adult male kidney cDNA, RIKEN full-length enriched library, clone:0610012C11:homogentisate 1, 2-dioxygenase, full insert sequence. |
| 1454067_a_at | 103.8 | P | 264.7 | 1.5 | I | P | 240.1 | P | 108.9 | P | -1.1 | D | 4931406C07Rik | gb:AK016432.1 /DB_XREF=gi:12855157 /FEA=mRNA /CNT=1 /TID=Mm.182227.2 /TIER=ConsEnd /STK=0 /UG=Mm.182227 /LL=70984 /UG_GENE=4931406C07Rik /UG_TITLE=RIKEN cDNA 4931406C07 gene /DEF=Mus musculus adult male testis cDNA, RIKEN full-length enriched library, clone:4931406C07:homolog to HYPOTHETICAL 35.1 KDA PROTEIN, full insert sequence. |
| 1454638_a_at | 1191.5 | P | 2798 | 1.2 | I | P | 6888.2 | P | 833.4 | P | -2.9 | D | Pah | gb:AW106920 /DB_XREF=gi:6077720 /DB_XREF=um34c04.x1 /CLONE=IMAGE:2236422 /FEA=EST /CNT=238 /TID=Mm.2422.2 /TIER=Stack /STK=106 /UG=Mm.2422 /LL=18478 /UG_GENE=Pah /UG_TITLE=phenylalanine hydroxylase |
| 1456306_a_at | 309.9 | P | 522.5 | 0.8 | I | P | 6490.1 | P | 3242.1 | P | -1 | D | Umod | gb:BB747266 /DB_XREF=gi:16150203 /DB_XREF=BB747266 /CLONE=F530201M24 /FEA=EST /CNT=12 /TID=Mm.10826.2 /TIER=Stack /STK=12 /UG=Mm.10826 /LL=22242 /UG_GENE=Umod /UG_TITLE=uromodulin |
| 1456388_at | 864.8 | P | 1134.9 | 0.5 | I | P | 1913.2 | P | 1114.3 | P | -1 | D | AU040868 | gb:AV378604 /DB_XREF=gi:16398189 /DB_XREF=AV378604 /CLONE=9130422H11 /FEA=EST /CNT=103 /TID=Mm.203947.1 /TIER=Stack /STK=10 /UG=Mm.203947 /LL=105982 /UG_GENE=AU040868 /UG_TITLE=expressed sequence AU040868 |
| 1456748_a_at | 104.8 | A | 187.7 | 0.5 | I | P | 282.7 | P | 126.4 | A | -1.1 | D | Nipsnap1 | gb:AV306253 /DB_XREF=gi:6338767 /DB_XREF=AV306253 /CLONE=5730537C13 /FEA=EST /CNT=8 /TID=Mm.16958.2 /TIER=Stack /STK=8 /UG=Mm.16958 /LL=18082 /UG_GENE=Nipsnap1 /UG_TITLE=4-nitrophenylphosphatase domain and non-neuronal SNAP25-like protein homolog 1 (C. elegans) |
| 1459917_at | 68.4 | A | 316.4 | 2 | I | P | 224.2 | P | 154.9 | P | -0.7 | D | C75939 | gb:BG078867 /DB_XREF=gi:12561435 /DB_XREF=H3033A04-5 /CLONE=H3033A04 /FEA=EST /CNT=6 /TID=Mm.25583.1 /TIER=ConsEnd /STK=0 /UG=Mm.25583 /LL=97681 /UG_GENE=C75939 /UG_TITLE=expressed sequence C75939 |
| 1460167_at | 359.4 | P | 521.2 | 0.5 | I | P | 731.6 | P | 360.6 | P | -0.8 | D | D18Wsu181e | gb:BC012407.1 /DB_XREF=gi:15214564 /FEA=FLmRNA /CNT=262 /TID=Mm.30250.1 /TIER=FL+Stack /STK=175 /UG=Mm.30250 /LL=28121 /UG_GENE=D18Wsu181e /DEF=Mus musculus, clone MGC:18699 IMAGE:4191228, mRNA, complete cds. /PROD=Unknown (protein for MGC:18699) /FL=gb:BC012407.1 gb:NM_138600.1 |
| 1460244_at | 287.6 | P | 550.4 | 0.8 | I | P | 461.1 | P | 188.5 | A | -1.4 | D | AI195023 | gb:NM_133995.1 /DB_XREF=gi:19527211 /GEN=AI195023 /FEA=FLmRNA /CNT=52 /TID=Mm.25557.1 /TIER=FL+Stack /STK=17 /UG=Mm.25557 /LL=103149 /DEF=Mus musculus expressed sequence AI195023 (AI195023), mRNA. /PROD=expressed sequence AI195023 /FL=gb:BC021388.1 gb:NM_133995.1 |

Supplementary Table S2. Genes up regulated in male and down regulated in female sfx mice.

| gene aff id | Balb-F-bone_Signal | Balb-F-bone_Detection | Sfx-F-bone_Signal | Balb-F-bone_Signal Log Ratio | Balb-F-bone_Change | Sfx-F-bone_Detection | Balb-M-bone_Signal | Balb-M-bone_Detection | Sfx-M-bone_Signal | Sfx-M-bone_Detection | Sfx-M-bone_Signal Log Ratio | Sfx-M-bone_Change | Descriptions | | Detailed description |
| --- | --- | --- | --- | --- | --- | --- | --- | --- | --- | --- | --- | --- | --- | --- | --- |
| 1415728_at | 1012.3 | P | 412.5 | -0.9 | D | P | 397.5 | P | 680.6 | P | 0.7 | I | Pabpn1 | gb:BG806432 /DB_XREF=gi:17953380 /DB_XREF=2024-83 /FEA=EST /CNT=98 /TID=Mm.7723.2 /TIER=Stack /STK=41 /UG=Mm.7723 /LL=54196 /UG_GENE=Pabpn1 /UG_TITLE=poly(A) binding protein, nuclear 1 | |
| 1415888_at | 1311.5 | P | 830.8 | -0.8 | D | P | 818.3 | P | 1157.9 | P | 0.4 | I | Hdgf | gb:NM_008231.1 /DB_XREF=gi:6680196 /GEN=Hdgf /FEA=FLmRNA /CNT=500 /TID=Mm.1141.1 /TIER=FL+Stack /STK=182 /UG=Mm.1141 /LL=15191 /DEF=Mus musculus hepatoma-derived growth factor (Hdgf), mRNA. /PROD=hepatoma-derived growth factor /FL=gb:D63707.1 gb:AF251787.1 gb:BC021654.1 gb:NM_008231.1 gb:BC005713.1 | |
| 1416101_a_at | 5804.1 | P | 2957.9 | -0.8 | D | P | 3200.1 | P | 5052.7 | P | 0.6 | I | H1f2 | gb:NM_015786.1 /DB_XREF=gi:9845256 /GEN=H1f2 /FEA=FLmRNA /CNT=188 /TID=Mm.193539.1 /TIER=FL+Stack /STK=127 /UG=Mm.193539 /LL=50708 /DEF=Mus musculus H1 histone family, member 2 (H1f2), mRNA. /PROD=H1 histone family, member 2 /FL=gb:NM_015786.1 | |
| 1416359_at | 565.8 | P | 56.8 | -2.3 | D | P | 539.3 | P | 612.9 | P | 0.3 | I | Snag1 | gb:NM_130796.1 /DB_XREF=gi:18644889 /GEN=Snag1 /FEA=FLmRNA /CNT=140 /TID=Mm.33721.1 /TIER=FL+Stack /STK=95 /UG=Mm.33721 /LL=170625 /DEF=Mus musculus sorting nexin associated golgi protein 1 (Snag1), mRNA. /PROD=sorting nexin associated golgi protein 1 /FL=gb:NM_130796.1 gb:AF408408.1 | |
| 1416906_at | 5121.3 | P | 3704.9 | -0.4 | D | P | 3677.5 | P | 4236.2 | P | 0.2 | I | Anapc5 | gb:NM_021505.1 /DB_XREF=gi:10946919 /GEN=Anapc5 /FEA=FLmRNA /CNT=258 /TID=Mm.45312.1 /TIER=FL+Stack /STK=64 /UG=Mm.45312 /LL=59008 /DEF=Mus musculus anaphase-promoting complex subunit 5 (Anapc5), mRNA. /PROD=anaphase-promoting complex subunit 5 /FL=gb:NM_021505.1 | |
| 1417013_at | 2006 | P | 769.8 | -1.5 | D | P | 1042.1 | P | 1234.4 | P | 0.3 | I | Cryac | gb:NM_030704.1 /DB_XREF=gi:13507645 /GEN=Cryac /FEA=FLmRNA /CNT=159 /TID=Mm.21549.1 /TIER=FL+Stack /STK=60 /UG=Mm.21549 /LL=80888 /DEF=Mus musculus crystallin, alpha C (Cryac), mRNA. /PROD=heat shock protein 20-like protein /FL=gb:BC011219.1 gb:NM_030704.1 gb:AF250139.1 gb:AF273453.1 | |
| 1417522_at | 462.3 | P | 309 | -0.7 | D | P | 233.7 | P | 824.3 | P | 1.9 | I | Fbxo32 | gb:NM_026346.1 /DB_XREF=gi:13385847 /GEN=Fbxo32 /FEA=FLmRNA /CNT=90 /TID=Mm.40466.1 /TIER=FL+Stack /STK=43 /UG=Mm.40466 /LL=67731 /DEF=Mus musculus f-box only protein 32 (Fbxo32), mRNA. /PROD=f-box only protein 32 /FL=gb:AF441120.1 gb:NM_026346.1 gb:BC027211.1 | |
| 1417654_at | 1244.9 | P | 170.1 | -2.5 | D | P | 438.1 | P | 839.3 | P | 0.4 | I | Sdc4 | gb:NM_011521.1 /DB_XREF=gi:6755441 /GEN=Sdc4 /FEA=FLmRNA /CNT=177 /TID=Mm.3815.1 /TIER=FL+Stack /STK=39 /UG=Mm.3815 /LL=20971 /DEF=Mus musculus syndecan 4 (Sdc4), mRNA. /PROD=syndecan 4 /FL=gb:D89571.1 gb:NM_011521.1 gb:BC005679.1 | |
| 1417879_at | 980.9 | P | 465.6 | -1.1 | D | P | 607.3 | P | 812.5 | P | 0.3 | I | 1110060M21Rik | gb:NM_025424.1 /DB_XREF=gi:13384817 /GEN=1110060M21Rik /FEA=FLmRNA /CNT=49 /TID=Mm.46444.1 /TIER=FL+Stack /STK=34 /UG=Mm.46444 /LL=66208 /DEF=Mus musculus RIKEN cDNA 1110060M21 gene (1110060M21Rik), mRNA. /PROD=RIKEN cDNA 1110060M21 /FL=gb:NM_025424.1 | |
| 1418702_a_at | 1400 | P | 640.7 | -0.7 | D | P | 965.8 | P | 1086.6 | P | 0.3 | I | 2810428I15Rik | gb:NM_025577.1 /DB_XREF=gi:13385019 /GEN=2810428I15Rik /FEA=FLmRNA /CNT=48 /TID=Mm.28242.1 /TIER=FL+Stack /STK=19 /UG=Mm.28242 /LL=66462 /DEF=Mus musculus RIKEN cDNA 2810428I15 gene (2810428I15Rik), mRNA. /PROD=RIKEN cDNA 2810428I15 /FL=gb:NM_025577.1 | |
| 1418755_at | 1489.3 | P | 917.9 | -0.9 | D | P | 983 | P | 1287.2 | P | 0.2 | I | Tbx15 | gb:NM_009323.1 /DB_XREF=gi:6678228 /GEN=Tbx15 /FEA=FLmRNA /CNT=24 /TID=Mm.10797.1 /TIER=FL+Stack /STK=19 /UG=Mm.10797 /LL=21384 /DEF=Mus musculus T-box 15 (Tbx15), mRNA. /PROD=T-box 15 /FL=gb:NM_009323.1 gb:AF041822.1 | |
| 1419150_at | 860.8 | P | 206.4 | -1.5 | D | P | 516.3 | P | 705 | P | 0.6 | I | Myf6 | gb:NM_008657.1 /DB_XREF=gi:6678983 /GEN=Myf6 /FEA=FLmRNA /CNT=14 /TID=Mm.11.1 /TIER=FL+Stack /STK=13 /UG=Mm.11 /LL=17878 /DEF=Mus musculus myogenic factor 6 (Myf6), mRNA. /PROD=myogenic factor 6 /FL=gb:NM_008657.1 | |
| 1419839_x_at | 2230.9 | P | 1511.8 | -0.5 | D | P | 1165.3 | P | 1447.4 | P | 0.2 | I | D19Wsu55e | gb:AA409562 /DB_XREF=gi:2066249 /DB_XREF=EST01334 /CLONE=C0008F07 /FEA=EST /CNT=1 /TID=Mm.218762.1 /TIER=ConsEnd /STK=1 /UG=Mm.218762 /LL=28000 /UG_GENE=D19Wsu55e /UG_TITLE=DNA segment, Chr 19, Wayne State University 55, expressed | |
| 1419933_at | 38.4 | P | 32.8 | -1 | D | A | 0.6 | A | 43.2 | A | 2.5 | I | AV006589 | gb:AV006589 /DB_XREF=gi:4783576 /DB_XREF=AV006589 /CLONE=1100006A23 /FEA=EST /CNT=1 /TID=Mm.198379.1 /TIER=ConsEnd /STK=1 /UG=Mm.198379 /LL=99251 /UG_GENE=AV006589 /UG_TITLE=expressed sequence AV006589 | |
| 1420884_at | 2464.4 | P | 1542.7 | -0.6 | D | P | 1343.4 | P | 5232.1 | P | 2 | I | 2310045A07Rik | gb:AK008863.1 /DB_XREF=gi:12843311 /FEA=FLmRNA /CNT=90 /TID=Mm.29132.1 /TIER=Stack /STK=43 /UG=Mm.29132 /LL=66402 /UG_GENE=2310045A07Rik /UG_TITLE=RIKEN cDNA 2310045A07 gene /DEF=Mus musculus adult male stomach cDNA, RIKEN full-length enriched library, clone:2210408O14:homolog to SARCOLIPIN, full insert sequence. /FL=gb:NM_025540.1 | |
| 1422807_at | 1136.9 | P | 639.8 | -0.8 | D | P | 438.3 | P | 912 | P | 0.8 | I | Arf5 | gb:NM_007480.1 /DB_XREF=gi:6680721 /GEN=Arf5 /FEA=FLmRNA /CNT=31 /TID=Mm.4996.1 /TIER=FL+Stack /STK=11 /UG=Mm.4996 /LL=11844 /DEF=Mus musculus ADP-ribosylation factor 5 (Arf5), mRNA. /PROD=ADP-ribosylation factor 5 /FL=gb:D87902.1 gb:NM_007480.1 | |
| 1422813_at | 1018.2 | P | 277.2 | -2 | D | P | 801.2 | P | 1037.7 | P | 0.4 | I | Cacng1 | gb:NM_007582.1 /DB_XREF=gi:6671657 /GEN=Cacng1 /FEA=FLmRNA /CNT=28 /TID=Mm.57093.1 /TIER=FL+Stack /STK=11 /UG=Mm.57093 /LL=12299 /DEF=Mus musculus calcium channel, voltage-dependent, gamma subunit 1 (Cacng1), mRNA. /PROD=calcium channel, voltage-dependent, gammasubunit 1 /FL=gb:NM_007582.1 | |
| 1422993_s_at | 730.8 | P | 431.3 | -0.8 | D | P | 245.6 | P | 521 | P | 1.1 | I | Refbp2 | gb:NM_019484.1 /DB_XREF=gi:9507038 /GEN=Refbp2 /FEA=FLmRNA /CNT=12 /TID=Mm.10364.1 /TIER=FL /STK=0 /UG=Mm.10364 /LL=56009 /DEF=Mus musculus RNA and export factor binding protein 2 (Refbp2), mRNA. /PROD=RNA and export factor binding protein 2 /FL=gb:NM_019484.1 | |
| 1424028_at | 908.5 | P | 473.1 | -0.8 | D | P | 975.9 | P | 1346.4 | P | 0.5 | I | FLJ20400 | gb:BC023107.1 /DB_XREF=gi:18606228 /FEA=FLmRNA /CNT=143 /TID=Mm.28552.1 /TIER=FL+Stack /STK=60 /UG=Mm.28552 /DEF=Mus musculus, Similar to hypothetical protein FLJ20400, clone MGC:28104 IMAGE:3966700, mRNA, complete cds. /PROD=Similar to hypothetical protein FLJ20400 /FL=gb:BC023107.1 | |
| 1424130_a_at | 2283.1 | P | 1030.1 | -1.2 | D | P | 1177.3 | P | 2151.6 | P | 0.7 | I | 2310075E07Rik | gb:BC012674.1 /DB_XREF=gi:15215127 /FEA=FLmRNA /CNT=70 /TID=Mm.21864.1 /TIER=FL+Stack /STK=51 /UG=Mm.21864 /LL=69669 /UG_GENE=2310075E07Rik /DEF=Mus musculus, Similar to polymerase I and transcript release factor, clone MGC:13867 IMAGE:4206082, mRNA, complete cds. /PROD=Similar to polymerase I and transcript releasefactor /FL=gb:BC012674.1 | |
| 1424345_s_at | 937 | P | 516.6 | -0.9 | D | P | 504.4 | P | 750.8 | P | 0.8 | I | UBC12 | gb:BC021792.1 /DB_XREF=gi:18256833 /FEA=FLmRNA /CNT=117 /TID=Mm.196580.1 /TIER=FL+Stack /STK=36 /UG=Mm.196580 /DEF=Mus musculus, ubiquitin-conjugating enzyme E2M (UBC12 homolog, yeast), clone MGC:25453 IMAGE:4235190, mRNA, complete cds. /PROD=ubiquitin-conjugating enzyme E2M (UBC12 homolog,yeast) /FL=gb:BC021792.1 | |
| 1424454_at | 835.8 | P | 627.8 | -0.6 | D | P | 566 | P | 817.8 | P | 0.6 | I | DKFZP564G2022 | gb:BC027354.1 /DB_XREF=gi:20072714 /FEA=FLmRNA /CNT=83 /TID=Mm.171349.1 /TIER=FL+Stack /STK=31 /UG=Mm.171349 /DEF=Mus musculus, Similar to DKFZP564G2022 protein, clone MGC:28282 IMAGE:4010477, mRNA, complete cds. /PROD=Similar to DKFZP564G2022 protein /FL=gb:BC027354.1 gb:BC024676.1 | |
| 1424480_s_at | 1072 | P | 564.5 | -0.9 | D | P | 593.9 | P | 812.1 | P | 0.6 | I | Akt2 | gb:BC026151.1 /DB_XREF=gi:20072783 /FEA=FLmRNA /CNT=78 /TID=Mm.177194.2 /TIER=FL+Stack /STK=30 /UG=Mm.177194 /LL=11652 /UG_GENE=Akt2 /DEF=Mus musculus, thymoma viral proto-oncogene 2, clone MGC:14031 IMAGE:4187425, mRNA, complete cds. /PROD=thymoma viral proto-oncogene 2 /FL=gb:BC026151.1 | |
| 1426060_at | 904.8 | P | 485 | -1.1 | D | P | 2216.7 | P | 2939.8 | P | 0.4 | I | Tf subfamily, member 30 | gb:BC007159.1 /DB_XREF=gi:13938088 /FEA=FLmRNA /CNT=1 /TID=Mm.212861.1 /TIER=FL /STK=1 /UG=Mm.212861 /DEF=Mus musculus, Similar to L1 repeat, Tf subfamily, member 30, clone MGC:7372 IMAGE:3487559, mRNA, complete cds. /PROD=Similar to L1 repeat, Tf subfamily, member 30 /FL=gb:BC007159.1 | |
| 1426061_x_at | 1013.8 | P | 483.7 | -1.3 | D | P | 2289.4 | P | 3055.4 | P | 0.5 | I | Tf subfamily, member 30 | gb:BC007159.1 /DB_XREF=gi:13938088 /FEA=FLmRNA /CNT=1 /TID=Mm.212861.1 /TIER=FL /STK=1 /UG=Mm.212861 /DEF=Mus musculus, Similar to L1 repeat, Tf subfamily, member 30, clone MGC:7372 IMAGE:3487559, mRNA, complete cds. /PROD=Similar to L1 repeat, Tf subfamily, member 30 /FL=gb:BC007159.1 | |
| 1426690_a_at | 1195.5 | P | 820.9 | -0.6 | D | P | 666.2 | P | 866.8 | P | 0.1 | I | Srebf1 | gb:AI326423 /DB_XREF=gi:4060852 /DB_XREF=mm45e05.x1 /CLONE=IMAGE:524480 /FEA=mRNA /CNT=294 /TID=Mm.214958.1 /TIER=Stack /STK=113 /UG=Mm.214958 /LL=20787 /UG_GENE=Srebf1 /UG_TITLE=sterol regulatory element binding factor 1 | |
| 1426710_at | 1886.4 | P | 914.7 | -1.1 | D | P | 539.4 | P | 760.5 | P | 0.4 | I | Calm3 | gb:BB396904 /DB_XREF=gi:16411476 /DB_XREF=BB396904 /CLONE=C230099N07 /FEA=mRNA /CNT=175 /TID=Mm.1147.1 /TIER=Stack /STK=98 /UG=Mm.1147 /UG_TITLE=Mus musculus calmodulin III (Calm3) mRNA, 3 untranslated region | |
| 1427263_at | 445.5 | P | 110.1 | -2 | D | P | 4.8 | A | 222.5 | P | 3.9 | I | Xist | gb:R74734 /DB_XREF=gi:849937 /DB_XREF=MDB0905 /FEA=mRNA /CNT=151 /TID=Mm.4095.1 /TIER=ConsEnd /STK=1 /UG=Mm.4095 /LL=22438 /UG_GENE=Xist /UG_TITLE=inactive X specific transcripts | |
| 1427346_at | 75.8 | P | 9.4 | -3 | D | A | 63.4 | A | 142.6 | P | 1.4 | I | Ott | gb:X96606.1 /DB_XREF=gi:1236762 /FEA=mRNA /CNT=18 /TID=Mm.3782.1 /TIER=ConsEnd /STK=6 /UG=Mm.3782 /LL=18422 /UG_GENE=Ott /DEF=M.musculus mRNA for Ott protein, clone ak27. /PROD=Ott protein | |
| 1427903_at | 2425.3 | P | 1337.3 | -0.7 | D | P | 1167.2 | P | 1544.1 | P | 0.2 | I | 1700008C22Rik | gb:BB650419 /DB_XREF=gi:16484674 /DB_XREF=BB650419 /CLONE=C230098K17 /FEA=mRNA /CNT=77 /TID=Mm.177990.1 /TIER=Stack /STK=37 /UG=Mm.177990 /LL=75454 /UG_GENE=1700008C22Rik /UG_TITLE=RIKEN cDNA 1700008C22 gene | |
| 1428465_at | 2787.5 | P | 2032.6 | -0.6 | D | P | 1736.2 | P | 2346.9 | P | 0.5 | I | 2010004E11Rik | gb:AK010168.1 /DB_XREF=gi:12845419 /FEA=mRNA /CNT=73 /TID=Mm.27499.1 /TIER=Stack /STK=40 /UG=Mm.27499 /LL=69804 /UG_GENE=2010004E11Rik /UG_TITLE=RIKEN cDNA 2010004E11 gene /DEF=Mus musculus adult male tongue cDNA, RIKEN full-length enriched library, clone:2310075E05:homolog to SEVEN TRANSMEMBRANE DOMAIN PROTEIN, full insert sequence. | |
| 1434135_at | 628.6 | P | 340.8 | -0.9 | D | P | 290.1 | P | 421 | P | 0.7 | I | C80633 | gb:BB107552 /DB_XREF=gi:15407988 /DB_XREF=BB107552 /CLONE=9530006I10 /FEA=EST /CNT=48 /TID=Mm.21686.2 /TIER=Stack /STK=41 /UG=Mm.21686 /LL=97884 /UG_GENE=C80633 /UG_TITLE=expressed sequence C80633 | |
| 1434507_at | 1649.6 | P | 941.6 | -1 | D | P | 1084.9 | P | 1504.3 | P | 0.4 | I | C85514 | gb:BG076209 /DB_XREF=gi:12558778 /DB_XREF=H3156H07-3 /CLONE=H3156H07 /FEA=EST /CNT=49 /TID=Mm.27070.1 /TIER=Stack /STK=30 /UG=Mm.27070 /LL=97015 /UG_GENE=C85514 /UG_TITLE=expressed sequence C85514 | |
| 1436510_a_at | 1211.9 | P | 839.7 | -0.6 | D | P | 1058.9 | P | 1365.2 | P | 0.2 | I | Lrrfip2 | gb:BE456782 /DB_XREF=gi:9478216 /DB_XREF=up94f07.x1 /CLONE=IMAGE:2797573 /FEA=EST /CNT=28 /TID=Mm.29797.2 /TIER=Stack /STK=8 /UG=Mm.29797 /LL=71268 /UG_GENE=Lrrfip2 /UG_TITLE=leucine rich repeat (in FLII) interacting protein 2 | |
| 1436936_s_at | 4740.3 | P | 2600 | -1.1 | D | P | 164.5 | P | 2101.3 | P | 3.2 | I | Tsix | gb:BG806300 /DB_XREF=gi:17953245 /DB_XREF=2022-96 /FEA=EST /CNT=67 /TID=Mm.197672.2 /TIER=Stack /STK=34 /UG=Mm.197672 /LL=22097 /UG_GENE=Tsix /UG_TITLE=X (inactive)-specific transcript, antisense | |
| 1437194_x_at | 1587.4 | P | 1011.5 | -0.6 | D | P | 1281.1 | P | 1728 | P | 0.5 | I | 1200011O22Rik | gb:BB252479 /DB_XREF=gi:8945225 /DB_XREF=BB252479 /CLONE=A730051K16 /FEA=EST /CNT=23 /TID=Mm.3094.2 /TIER=Stack /STK=23 /UG=Mm.3094 /LL=71735 /UG_GENE=1200011O22Rik /UG_TITLE=RIKEN cDNA 1200011O22 gene | |
| 1437280_s_at | 9617.1 | P | 6038.3 | -0.5 | D | P | 6150.1 | P | 7082.4 | P | 0.2 | I | 1200009K13Rik | gb:AV110762 /DB_XREF=gi:5264842 /DB_XREF=AV110762 /CLONE=2600014D09 /FEA=EST /CNT=21 /TID=Mm.18700.2 /TIER=Stack /STK=21 /UG=Mm.18700 /LL=66870 /UG_GENE=1200009K13Rik /UG_TITLE=RIKEN cDNA 1200009K13 gene | |
| 1437867_at | 350.3 | P | 52.3 | -2.3 | D | A | 573.8 | P | 677 | P | 0.5 | I | Cd84 | gb:C77655 /DB_XREF=gi:2517985 /DB_XREF=C77655 /CLONE=J0035E10 /FEA=EST /CNT=30 /TID=Mm.21882.5 /TIER=Stack /STK=13 /UG=Mm.21882 /LL=12523 /UG_GENE=Cd84 /UG_TITLE=CD84 antigen | |
| 1438385_s_at | 1148.7 | P | 839.4 | -0.5 | D | P | 1184.2 | P | 1452 | P | 0.3 | I | 0610012D09Rik | gb:BB068040 /DB_XREF=gi:8525454 /DB_XREF=BB068040 /CLONE=8030469I16 /FEA=EST /CNT=11 /TID=Mm.29122.5 /TIER=Stack /STK=11 /UG=Mm.29122 /LL=59052 /UG_GENE=0610012D09Rik /UG_TITLE=RIKEN cDNA 0610012D09 gene | |
| 1448262_at | 1379.1 | P | 811.8 | -0.7 | D | P | 596.6 | P | 1185.3 | P | 1 | I | Psmb2 | gb:NM_011970.1 /DB_XREF=gi:6755199 /GEN=Psmb2 /FEA=FLmRNA /CNT=195 /TID=Mm.22233.1 /TIER=FL+Stack /STK=110 /UG=Mm.22233 /LL=26445 /DEF=Mus musculus proteasome (prosome, macropain) subunit, beta type 2 (Psmb2), mRNA. /PROD=proteasome (prosome, macropain) subunit, betatype, 2 /FL=gb:BC008265.1 gb:AF060090.1 gb:NM_011970.1 | |
| 1449002_at | 2534.1 | P | 1428.5 | -0.9 | D | P | 1551.1 | P | 2212.1 | P | 0.7 | I | Phlda3 | gb:NM_013750.1 /DB_XREF=gi:7305376 /GEN=Phlda3 /FEA=FLmRNA /CNT=99 /TID=Mm.34346.1 /TIER=FL+Stack /STK=27 /UG=Mm.34346 /LL=27280 /DEF=Mus musculus pleckstrin homology-like domain, family A, member 3 (Phlda3), mRNA. /PROD=pleckstrin homology-like domain, family A,member 3 /FL=gb:NM_013750.1 gb:BC023408.1 | |
| 1449018_at | 6678.6 | P | 4683.2 | -0.6 | D | P | 2577.3 | P | 5686.9 | P | 1.1 | I | Pfn1 | gb:NM_011072.1 /DB_XREF=gi:6755039 /GEN=Pfn1 /FEA=FLmRNA /CNT=168 /TID=Mm.2647.1 /TIER=FL+Stack /STK=26 /UG=Mm.2647 /LL=18643 /DEF=Mus musculus profilin 1 (Pfn1), mRNA. /PROD=profilin 1 /FL=gb:BC002080.1 gb:NM_011072.1 | |
| 1449027_at | 579.9 | P | 299.9 | -1 | D | P | 305.3 | P | 569 | P | 0.6 | I | Arhu | gb:NM_133955.1 /DB_XREF=gi:19923059 /GEN=Arhu /FEA=FLmRNA /CNT=85 /TID=Mm.28954.1 /TIER=FL+Stack /STK=26 /UG=Mm.28954 /LL=69581 /DEF=Mus musculus ras homolog gene family, member U (Arhu), mRNA. /PROD=ras homolog gene family, member U /FL=gb:NM_133955.1 gb:AB051827.1 gb:BC020060.1 gb:AF378088.1 | |
| 1451012_a_at | 4171.7 | P | 2203.7 | -0.7 | D | P | 2921.2 | P | 3856 | P | 0.3 | I | Csda | gb:AV216648 /DB_XREF=gi:6157493 /DB_XREF=AV216648 /CLONE=2410165I03 /FEA=FLmRNA /CNT=234 /TID=Mm.193526.1 /TIER=Stack /STK=15 /UG=Mm.193526 /LL=56449 /UG_GENE=Csda /UG_TITLE=cold shock domain protein A /FL=gb:D14485.1 gb:AF248547.1 gb:NM_019638.1 | |
| 1451290_at | 1964.9 | P | 951.8 | -1 | D | P | 980.6 | P | 1512.6 | P | 0.6 | I | MAP1 | gb:BC010596.1 /DB_XREF=gi:14714881 /FEA=FLmRNA /CNT=95 /TID=Mm.196239.1 /TIER=FL+Stack /STK=41 /UG=Mm.196239 /LL=66734 /UG_GENE=4922501H04Rik /DEF=Mus musculus, MAP1 light chain 3-like protein 1, clone MGC:6325 IMAGE:3256801, mRNA, complete cds. /PROD=MAP1 light chain 3-like protein 1 /FL=gb:BC010596.1 | |
| 1451745_a_at | 1894.1 | P | 1229.2 | -0.5 | D | P | 1487.8 | P | 1828.4 | P | 0.2 | I | 2700001K05Rik | gb:BC026751.1 /DB_XREF=gi:20071192 /FEA=FLmRNA /CNT=36 /TID=Mm.41486.1 /TIER=FL /STK=1 /UG=Mm.41486 /LL=70103 /UG_GENE=2700001K05Rik /DEF=Mus musculus, Similar to putative cyclin G1 interacting protein, clone MGC:25516 IMAGE:3153902, mRNA, complete cds. /PROD=Similar to putative cyclin G1 interactingprotein /FL=gb:BC026751.1 | |
| 1455482_at | 507.4 | P | 276 | -1.1 | D | P | 217 | P | 382.2 | P | 0.7 | I | Tnnt2 | gb:BQ175609 /DB_XREF=gi:20351101 /DB_XREF=UI-M-DJ2-bvx-m-04-0-UI.s1 /CLONE=UI-M-DJ2-bvx-m-04-0-UI /FEA=EST /CNT=86 /TID=Mm.632.6 /TIER=Stack /STK=12 /UG=Mm.632 /LL=21956 /UG_GENE=Tnnt2 /UG_TITLE=troponin T2, cardiac | |
| 1456133_x_at | 2516.1 | P | 1837.2 | -0.5 | D | P | 1952 | P | 2499 | P | 0.2 | I | Itgb5 | gb:BB543646 /DB_XREF=gi:9614869 /DB_XREF=BB543646 /CLONE=E130211M19 /FEA=EST /CNT=16 /TID=Mm.6424.4 /TIER=Stack /STK=15 /UG=Mm.6424 /LL=16419 /UG_GENE=Itgb5 /UG_TITLE=integrin beta 5 | |
| 1460198_a_at | 2811 | P | 1921.2 | -0.5 | D | P | 2911.5 | P | 3816.8 | P | 0.4 | I | Psmb3 | gb:BC014783.1 /DB_XREF=gi:15928627 /FEA=FLmRNA /CNT=156 /TID=Mm.21874.1 /TIER=FL+Stack /STK=58 /UG=Mm.21874 /LL=26446 /UG_GENE=Psmb3 /DEF=Mus musculus, proteasome (prosome, macropain) subunit, beta type 3, clone MGC:5639 IMAGE:3500033, mRNA, complete cds. /PROD=proteasome (prosome, macropain) subunit, betatype 3 /FL=gb:AF060092.1 gb:BC014783.1 gb:NM_011971.1 | |
| 1460548_a_at | 556.7 | P | 201.1 | -1.2 | D | P | 206.3 | P | 369.2 | P | 1 | I | Eral1 | gb:AI505281 /DB_XREF=gi:4403132 /DB_XREF=vp98g05.x1 /CLONE=IMAGE:1092824 /FEA=EST /CNT=65 /TID=Mm.21096.6 /TIER=Stack /STK=64 /UG=Mm.21096 /LL=57837 /UG_GENE=Eral1 /UG_TITLE=Era (G-protein)-like 1 (E. coli) | |
